# Effectiveness of the Low Fermentable Oligosaccharides, Disaccharides, Monosaccharides, and Polyols Diet for Patients With Irritable Bowel Syndrome Managed in Primary and Secondary Care: A Systematic Review and Meta-Analysis of Randomized Trials

**DOI:** 10.7759/cureus.117606

**Published:** 2026-10-07

**Authors:** Mohamad Ahmad M Alenezi, Jana Fayez A Alfayez, Joud Mohammed B Alkhidhr, Amasi Saad M Alruwaili, Almaha Mansour M Alanazi, Imtinan Abdullah S Alqarni, Nawa Farhan M Alanazi, Amaal Fayez M Alenezi, Ruba Olum K Alshammari, Zuhaa Shaher Dhahawi Alanazi, Ghadah Dhiyab H Alanazi, Nouf Sami H Alanazi, Sarah Sultan A Alanazi, Aryam Hamdan Y Alanazi, Noor Gamal Abdelhamid Aly Mohamed

**Affiliations:** 1 Department of Family Medicine, Maternity and Children Hospital in Arar, Arar, SAU; 2 College of Medicine, Northern Border University, Arar, SAU; 3 College of Medicine, ‏King Saud University, Riyadh, SAU

**Keywords:** abdominal pain, fodmap diet, irritable bowel syndrome, meta-analysis, quality of life

## Abstract

The low fermentable oligosaccharides, disaccharides, monosaccharides, and polyols (FODMAP) diet has emerged as a promising dietary intervention for managing irritable bowel syndrome (IBS) symptoms. We aimed to evaluate the effectiveness of the low FODMAP diet compared with various control conditions in adults with IBS. The pre-specified primary outcome was the Irritable Bowel Syndrome Symptom Severity Score, whereas adequate relief, abdominal pain, bloating, and quality of life (QoL) were the secondary outcomes. A systematic search identified English-language randomized controlled trials (RCTs) from inception through 20 May 2026 that compared the low FODMAP diet with control conditions in adults with IBS. Statistical analyses were performed using RevMan 5.4 (Cochrane Collaboration, London, UK), with treatment effects expressed as standardized mean differences (SMDs) or odds ratios (ORs) with 95% confidence intervals (CIs). Heterogeneity was quantified using the I² statistic, and random-effects models were used for the primary analyses, with fixed-effect sensitivity analyses when heterogeneity was low. Twenty-three RCTs, encompassing 2,164 participants (1,852 completers), were included. Compared with traditional dietary advice, the low FODMAP diet significantly reduced IBS symptom severity score (SMD: −0.71; 95% CI (−1.02, −0.40)), improved adequate relief (OR: 2.15; 95% CI (1.54, 3.01)), reduced abdominal pain (SMD: −0.35; 95% CI (−0.54, −0.16)), decreased bloating (SMD: −0.45; 95% CI (−0.75, −0.15)), and improved QoL (SMD: 0.48; 95% CI (0.27, 0.69)). Similar benefits were observed against habitual diet comparators. Compared with other active dietary interventions, the low FODMAP diet did not significantly improve IBS symptom severity, adequate relief, or QoL, although it did modestly improve bloating. The low FODMAP diet is an effective intervention for reducing global IBS symptoms and improving QoL compared with traditional dietary advice and habitual diet; however, it does not appear to offer greater benefit than other structured active dietary interventions, aside from this modest, comparator-independent improvement in bloating. These findings suggest that structured dietary intervention and dietary support may contribute substantially to symptom improvement beyond FODMAP restriction itself.

## Introduction and background

Irritable bowel syndrome (IBS) is one of the most prevalent disorders of gut-brain interaction, affecting between 4% and 10% of the global adult population according to the Rome IV diagnostic criteria [1,2]. It is characterized by recurrent abdominal pain occurring at least one day per week, associated with alterations in stool frequency, stool form, or both, in the absence of detectable structural or biochemical abnormality. The Rome IV criteria, which represent the current gold standard for diagnosis, classify IBS into four subtypes based on predominant bowel habit: IBS with constipation (IBS-C), IBS with diarrhea (IBS-D), IBS with mixed bowel habits (IBS-M), and unclassified IBS (IBS-U) [3]. Although IBS is not associated with excess mortality, it imposes a disproportionate burden on patients’ health-related quality of life (QoL), occupational productivity, and psychological well-being, as well as substantial direct and indirect costs to healthcare systems worldwide [4,5].

IBS is a multifactorial disorder in which altered gut-brain communication, heightened gut sensitivity, altered motility, and changes in the gut microbiota are all thought to contribute [6,7]. Most IBS patients report that symptoms are exacerbated by dietary intake, with food identified as a key trigger in up to 84% of cases [8]. This observation has generated considerable scientific and clinical interest in dietary manipulation as a therapeutic strategy. It has provided the rationale for investigating specific dietary interventions, among which the low fermentable oligosaccharides, disaccharides, monosaccharides, and polyols (FODMAP) diet has emerged as the most extensively studied [9].

FODMAPs are a heterogeneous group of short-chain carbohydrates that share two key physiological properties: poor absorption in the small intestine and rapid fermentation by colonic microbiota [10]. These properties generate two principal pathophysiological effects. First, the osmotic activity of unabsorbed FODMAPs draws water into the intestinal lumen, increasing luminal volume and distension [11]. Second, rapid bacterial fermentation produces hydrogen and methane gas, further distending the bowel [12]. In individuals with IBS, who characteristically exhibit visceral hypersensitivity, these otherwise physiologically normal events are experienced as disproportionately painful and distressing, culminating in the hallmark symptoms of abdominal pain, bloating, distension, and altered defecation [11,13].

The low FODMAP diet is a structured, three-phase dietary intervention [14]. The first phase restricts high-FODMAP foods for two to six weeks to control symptoms. The second phase involves systematically reintroducing individual FODMAP subgroups to identify personal tolerance thresholds. The third and final phase involves personalizing the long-term diet, allowing the patient to consume tolerated foods and restricting only the specific FODMAPs that provoke symptoms [13,14]. A trained dietitian should deliver the diet, and the British Society of Gastroenterology, the American College of Gastroenterology, and other major clinical guideline bodies endorse it as a second-line dietary intervention [15,16].

Despite the growing evidence, important questions remain about the consistency and magnitude of low FODMAP diet benefits across different clinical comparators and outcome domains. In particular, the relative efficacy of the low FODMAP diet compared with other structured active dietary interventions, rather than passive or habitual diet controls, remains uncertain, with some trials showing no statistically significant between-group differences. Furthermore, prior meta-analyses have not systematically stratified pooled analyses by comparator type, limiting the interpretability of findings for clinicians practicing in both primary and secondary care settings. Here, primary care refers to first-contact community care (e.g., general practitioners), where IBS is usually diagnosed and first managed with general lifestyle and dietary advice, whereas secondary care refers to specialist gastroenterology and dietetic services to which patients are referred when symptoms persist; the distinction matters because access to dietitian-led low FODMAP delivery and the type of control advice differ between these settings.

Against this background, the present systematic review and meta-analysis was conducted to provide a comprehensive, comparator-stratified evaluation of the effectiveness of the low FODMAP diet in adults with IBS across five clinically relevant outcome domains (symptom severity measured by the Irritable Bowel Syndrome Symptom Severity Score (IBS-SSS), adequate relief, abdominal pain, bloating, and health-related QoL). By synthesizing evidence from 23 randomized controlled trials (RCTs) and 2,164 randomized participants, this review aims to generate insights that are directly applicable to dietary management decisions in both primary and secondary care.

## Review

Methodology

The conduct and reporting of this systematic review and meta-analysis followed the principles of the Cochrane Handbook for Systematic Reviews of Interventions, version 6, and the Preferred Reporting Items for Systematic Reviews and Meta-Analyses (PRISMA) guidelines [17].

Research question and objectives

The central research question guiding this systematic review and meta-analysis was: in adults with IBS managed in primary or secondary care, is the low FODMAP diet more effective than comparator dietary conditions, including traditional dietary advice, habitual diet, higher FODMAP diet, and other active dietary interventions not aligned with National Institute for Health and Care Excellence (NICE) guidelines?

The objectives were to evaluate the overall and comparator-stratified effectiveness of the low FODMAP diet in adults with IBS across five outcome domains: IBS symptom severity (IBS-SSS; primary outcome), adequate relief or global symptom response, abdominal pain severity, bloating or distension severity, and health-related QoL. Dichotomous outcomes were analyzed using ORs and continuous outcomes using SMDs, with analyses stratified by traditional dietary advice, habitual diet, a higher FODMAP diet, and other active dietary interventions not aligned with NICE guidelines. A secondary objective was to assess the risk of bias in the included RCTs and quantify statistical heterogeneity to contextualize the certainty of the synthesized evidence.

Inclusion criteria

Population

Eligible trials enrolled adult participants, defined as individuals aged 18 years or older, with a confirmed diagnosis of IBS established using recognized symptom-based diagnostic criteria, including the Rome II, Rome III, or Rome IV criteria. Trials enrolling participants diagnosed exclusively by clinician judgment without reference to validated criteria were excluded. Participants had to have IBS as their primary gastrointestinal condition, and we excluded studies if the enrolled population had a concurrent or overlapping organic gastrointestinal disease, including inflammatory bowel disease (Crohn’s disease or ulcerative colitis), coeliac disease, microscopic colitis, or other structural gastrointestinal pathology. We excluded trials that enrolled mixed populations of IBS and non-IBS participants unless IBS-specific data were reported separately and could be extracted in isolation. No restriction was placed on IBS subtype (IBS-C, IBS-D, IBS-M, or IBS-U), disease severity, sex, ethnicity, or clinical setting, thereby encompassing participants managed across both primary and secondary care.

Intervention

The intervention of interest was a structured low FODMAP diet, defined as a dietary program that restricts fermentable oligosaccharides, disaccharides, monosaccharides, and polyols to reduce gastrointestinal symptom burden in IBS. The low FODMAP diet could be delivered in any format, including individual dietitian-led consultations, group education sessions, written resources, or app-based guidance. It could be implemented as a full restriction phase, a combined restriction and reintroduction program, or a personalized long-term dietary plan, provided that the initial FODMAP-restricting phase was the focus of the comparison. Trials were included regardless of intervention duration, the degree of FODMAP restriction, or whether the intervention was supplemented with additional components such as probiotics or fiber, provided that the low FODMAP dietary component remained the primary experimental condition. It was compared against a defined control or comparator arm.

Comparators

The comparator conditions in this review reflected the range of control arms used across the eligible trial literature and were classified into four pre-specified subgroups for stratified meta-analysis. The first subgroup comprised trials comparing the low FODMAP diet to traditional dietary advice, defined as structured dietary guidance aligned with national IBS dietary recommendations, such as those issued by NICE or equivalent bodies. The second subgroup comprised trials comparing the low FODMAP diet to a habitual or usual diet, in which participants in the control arm received no specific dietary instruction and continued their existing dietary pattern. The third subgroup comprised trials comparing the low FODMAP diet to a high FODMAP diet, serving as a mechanistic comparator. The fourth subgroup comprised trials comparing the low FODMAP diet with other structured active dietary interventions, defined by the specific intervention delivered rather than by formal guideline alignment, including low-carbohydrate diets, gluten-free diets, and other structured dietary programs that differed from conventional dietary advice. For brevity, this subgroup is labeled throughout as "other active dietary interventions (non-NICE)"; the "non-NICE" designation is used only to distinguish this comparator from the NICE-aligned traditional dietary advice comprising the first subgroup and is not intended as a validated or reproducible clinical classification in its own right.

Outcomes

The five pre-specified outcomes were IBS-SSS (primary outcome), adequate relief or global symptom response, abdominal pain severity, bloating or distension severity, and health-related QoL; their definitions and analysis are described under measured outcomes.

Study Designs

This systematic review and meta-analysis included only RCTs. Both parallel-group and crossover RCTs were considered for inclusion; however, crossover trials were eligible only when data from the initial treatment period, that is, data collected prior to the crossover, were extractable and reported separately. Trials in which only post-crossover or combined data were reported, without the ability to isolate the primary period analysis, were excluded. No restriction was placed on trial duration, setting, country of conduct, or mode of dietary delivery, provided all other eligibility criteria were satisfied.

Exclusion criteria

Studies were excluded during the screening process on the basis of the following criteria. Non-randomized study designs, including cohort studies, case-control studies, uncontrolled before-after studies, case series, and narrative or systematic reviews, were not eligible. Trials conducted exclusively in pediatric populations, defined as participants under the age of 18 years, were excluded. Studies enrolling participants with a primary diagnosis of an organic gastrointestinal condition, such as inflammatory bowel disease or coeliac disease, were excluded even if a subset of participants also carried an IBS diagnosis, unless IBS-specific data were independently extractable. Crossover trials from which only pooled or post-crossover data were available, without a separable primary-period analysis, were excluded to avoid carry-over bias. Studies not published in the English language, as well as conference abstracts, letters, editorials, and unpublished datasets lacking sufficient methodological detail for quality appraisal and data extraction, were also excluded. Finally, trials that did not report any of the five pre-specified outcomes, IBS-SSS, adequate relief or global symptom response, abdominal pain severity, bloating or distension severity, or health-related QoL, in a format amenable to quantitative synthesis were excluded from the respective outcome-level meta-analyses.

Search strategy

Electronic Searches

The following databases, trial registries, and grey literature sources were searched for eligible studies: PubMed and MEDLINE (Ovid), Cochrane Central Register of Controlled Trials (CENTRAL), Web of Science, ProQuest, ClinicalTrials.gov, grey literature, ScienceDirect, and Scopus. The search included all English-language articles from inception to 20 May 2026.

The following search terms were used: ("irritable bowel syndrome" OR "IBS" OR "functional bowel disorder") AND ("low FODMAP" OR "FODMAP diet" OR "fermentable oligosaccharides" OR "fermentable carbohydrate*") AND ("symptom*" OR "abdominal pain" OR "bloating" OR "quality of life" OR "IBS-SSS") AND ("randomised controlled trial" OR "randomized controlled trial" OR "clinical trial").

Database-specific search strategies, including the date of the last search in each database and any filters applied, are provided in the Supplementary Table.

Other Resources

The first reviewer searched the reference lists of retrieved articles for other potentially relevant studies not identified through electronic searches.

Selection of studies

The first reviewer screened the retrieved reports for eligibility by title and abstract, then by full text. The second reviewer checked the retrieved studies, and the third reviewer resolved discrepancies through discussion.

Screening was therefore performed by one reviewer and verified by a second reviewer, with a third reviewer resolving disagreements, rather than as fully independent duplicate screening; this is acknowledged as a limitation. Multiple reports of the same trial were handled by including only the primary trial report; duplicate publications and secondary or sub-analyses of included trials were excluded (see Results, Study selection).

The Results section presents the study selection results, including the PRISMA flow diagram and reasons for exclusion at the full-text stage (Study selection).

Data extraction

The first reviewer extracted data from the included studies using a standardized data sheet. The extracted data included study ID (author, year), study design, intervention duration (FODMAP restriction, wks.), follow-up duration (wks.), country/setting, IBS diagnostic criteria, IBS subtype, total sample size (N), age (mean ± SD or median (IQR)), sex (% female), baseline symptom severity, intervention description (low FODMAP), comparator description, reintroduction phase included, concomitant treatments, extracted outcomes, symptom scale used, QoL scale used, and key results. The second reviewer independently checked all collected data for accuracy and completeness. Reviewers resolved disagreements through discussion and, if necessary, arbitration by a third reviewer.

Measured outcomes

The primary outcome was overall IBS symptom severity, measured using the validated IBS-SSS, a composite patient-reported instrument assessing the frequency and intensity of abdominal pain, bloating, bowel dissatisfaction, and interference with daily life. This outcome was analyzed as a continuous measure and pooled using SMD with 95% confidence intervals (CIs) to allow for cross-trial comparability.

The secondary outcomes comprised four additional domains. Adequate relief or global symptom response was defined as a positive patient-reported assessment of overall symptom improvement, regardless of the specific tool used across trials. It was analyzed as a dichotomous outcome using ORs with 95% CIs. Abdominal pain severity and bloating or abdominal distension severity were treated as continuous outcomes and pooled using SMDs. Health-related QoL was assessed using any validated QoL instrument applicable to IBS, including the IBS-QoL, SF-36, and EQ-5D, and was similarly pooled as a continuous outcome using SMDs, with a positive SMD indicating improvement in QoL favoring the low FODMAP diet.

Assessment of the risk of bias

The Cochrane RoB 2 tool (Cochrane Collaboration, London, UK) was used for RCTs. All the RoB figures were generated with the aid of the robvis package using R 4.3.2 software (R Core Team, Vienna, Austria).

Data synthesis

The study identification and selection process, including the full PRISMA flow and reasons for exclusion, is reported in the Results section (Study selection). The 23 included studies [8,12,18-38] were grouped into the four pre-specified comparator subgroups described under Comparators (traditional dietary advice, habitual diet, higher FODMAP diet, and other active dietary interventions), and outcome data for each subgroup were pooled separately for each of the five outcome domains (IBS-SSS, adequate relief, abdominal pain, bloating, and QoL). Crossover trials contributed only first-treatment-period data, as described under Study designs, to avoid potential carry-over effects. The statistical analysis below details effect measures, heterogeneity assessment, model selection, and sensitivity analyses.

Statistical analysis

All statistical analyses were performed using RevMan version 5.4. For continuous outcomes, treatment effects were expressed as SMDs with 95% CIs, given the variability in measurement scales across included studies. For dichotomous outcomes, treatment effects were expressed as ORs with 95% CIs using the Mantel-Haenszel method. Heterogeneity was assessed using the Chi² test and quantified using the I² statistic, with thresholds of <40%, 40% to <60%, 60%-75%, and >75% interpreted as low, moderate, substantial, and considerable heterogeneity, respectively. Random-effects models using the DerSimonian-Laird estimator were used as the primary approach for all pooled analyses, because clinical and methodological heterogeneity was anticipated a priori across trials that differed in populations, intervention delivery, and comparator conditions. For outcomes with low statistical heterogeneity (I² <40%), a fixed-effect model was additionally applied as a sensitivity analysis to assess the robustness of the pooled estimate to model choice. Sensitivity analyses were conducted by sequential exclusion of individual studies to explore the source of heterogeneity; these leave-one-out analyses were post hoc and not pre-specified, whereas the outcomes, comparator subgroups, and use of a random-effects model were pre-specified, and post hoc analyses are labeled as such in the Results and regarded as exploratory. Of the 2,164 randomized participants across included trials, 1,852 completed their assigned intervention; outcome data were analyzed on an available-case (completers) basis, that is, using the participants who completed the intervention and contributed follow-up data in each trial, with change-from-baseline data as reported in the original trial publications; the review authors did not impute missing outcome data, and the potential for attrition bias was appraised under the incomplete-outcome-data domain of RoB 2. A p-value <0.05 was considered statistically significant for all analyses. No adjustment for multiple comparisons was made, and borderline findings are interpreted as exploratory. Publication bias and small-study effects could not be formally assessed; Egger's test was not performed because no pooled comparison included 10 or more studies (maximum: seven), the minimum recommended in the Cochrane Handbook for reliable asymmetry detection and for Egger's regression to have adequate statistical power. Visual inspection of funnel plots was considered insufficient for meaningful interpretation, as apparent asymmetry with few studies may reflect between-study heterogeneity, small-study effects, or chance rather than true publication bias; accordingly, funnel plots are not presented. The certainty of evidence was not formally graded (e.g., with GRADE); risk of bias, heterogeneity, and imprecision are instead considered narratively in the Discussion. Heterogeneous structured dietary programs not aligned with NICE guidance were pooled into a single pre-specified comparator subgroup to allow comparator-stratified estimation against non-guideline diets; because this may combine clinically dissimilar interventions, estimates for this subgroup, particularly where heterogeneity was considerable (I² up to 95%) or few studies contributed, are interpreted cautiously.

Results

Study Selection

Electronic database searches identified 1,510 records. After removing 338 duplicate records and excluding records at the title/abstract screening stage, 345 records remained and were assessed for eligibility; 23 RCTs [8,12,18-38] met all inclusion criteria. The remaining studies were excluded based on predefined criteria; duplicate publications, secondary analyses, and sub-analyses of existing trials accounted for the largest proportion of exclusions (N = 144). Ineligible study designs, including reviews, meta-analyses, study protocols, non-randomized trials, qualitative studies, uncontrolled pre-post designs, and case reports, were the second most common reason (N = 59). Studies enrolling populations without confirmed IBS, including those with inflammatory bowel disease, coeliac disease, functional heartburn, gastro-esophageal reflux disease, functional dyspepsia, or healthy volunteers, were excluded (N = 35). Publications available only as conference abstracts or without sufficient data for extraction were also excluded (N = 21). Further exclusions were applied to crossover trials without separable primary-period data (N = 11), studies reporting irrelevant outcomes (N = 11), trials using non-dietary comparators such as pharmacotherapy or behavioral therapy (N = 9), inaccessible full texts (N = 9), trials comparing low FODMAP diet plus a supplement against low FODMAP diet alone without an independent dietary comparator (N = 6), studies in which the low FODMAP diet was not the primary intervention, including reintroduction, challenge, and dietary adherence app designs (N = 13), and post-hoc metabolomic or microbiological secondary analyses published in non-English languages (N = 4). Figure 1 summarizes the reasons for full-text exclusion [39].

**Figure 1:**
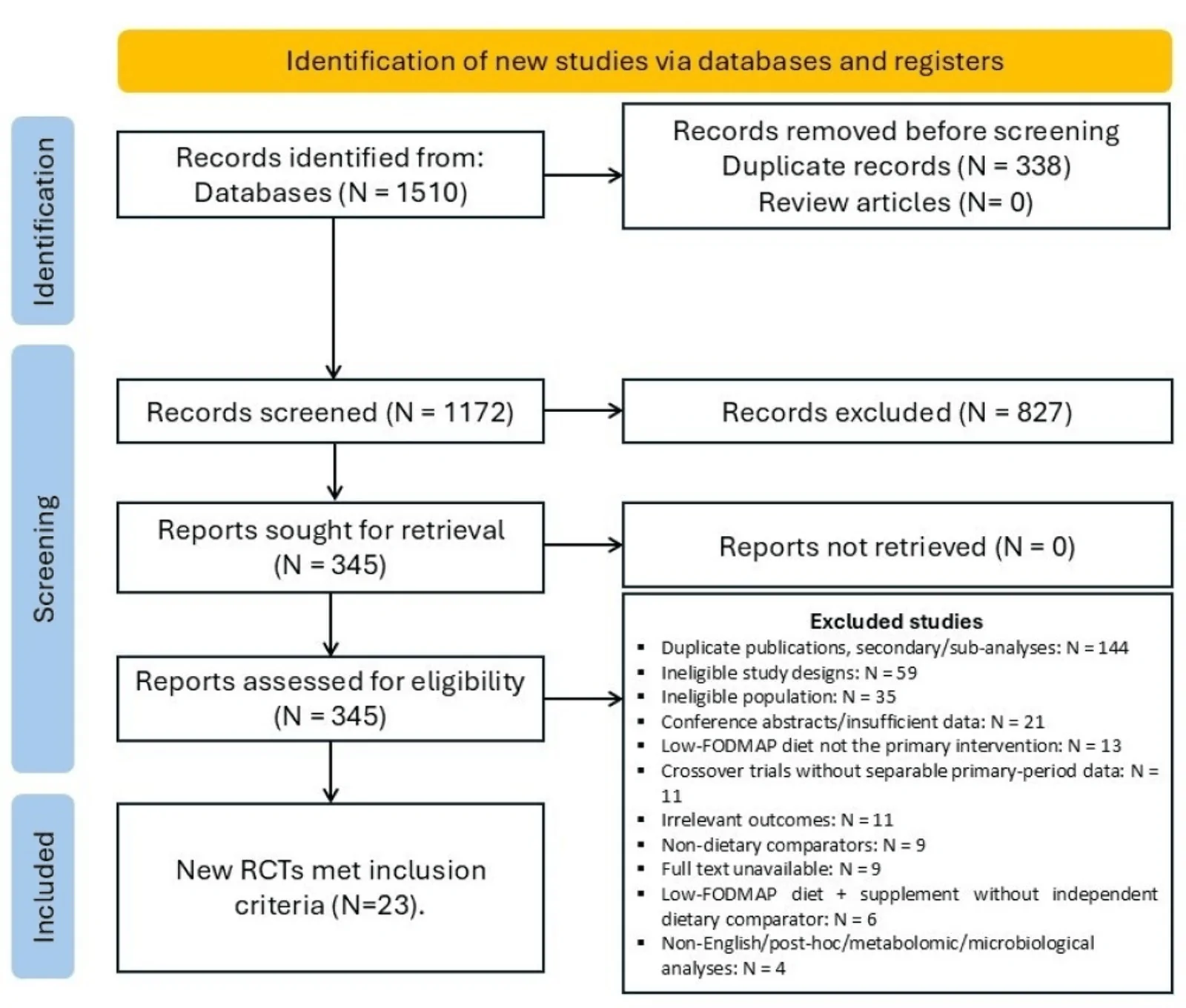
PRISMA flowchart RCT: randomized controlled trial, IBS: irritable bowel syndrome, FODMAP: fermentable oligosaccharides, disaccharides, monosaccharides, and polyols, PRISMA: Preferred Reporting Items for Systematic Reviews and Meta-Analyses

Characteristics of Included Studies

The main characteristics of the 23 included RCTs are summarized in Table 1, including study design, duration of the FODMAP restriction phase and follow-up, country and setting, IBS diagnostic criteria and subtype, sample size, participant age and sex, baseline symptom severity, intervention and comparator descriptions, and the outcomes and scales extracted. A total of 23 RCTs [8,12,18-38] were included in this meta-analysis, encompassing 2,164 randomized participants and 1,852 completed participants across all intervention arms. Sample sizes ranged from 29 to 155 participants per study. Across the included trials, participants were allocated to a range of comparator conditions, including traditional dietary advice, habitual diet, higher FODMAP diet, and other active dietary interventions not aligned with NICE, reflecting the variability of control conditions employed in the dietary management of IBS.

**Table 1:** Summary of included studies ACG: American College of Gastroenterology, AI: artificial intelligence, BIA: bioelectrical impedance analysis, BMI: body mass index, BRD: brief recommended diet, BSFS: Bristol Stool Form Scale (BSF: stool form), CFU: colony-forming units, CI: confidence interval, CPSS: Perceived Stress Scale, CRP: C-reactive protein, FDA: Food and Drug Administration, FFQ: Food Frequency Questionnaire, FODMAP: fermentable oligosaccharides, disaccharides, monosaccharides, and polyols, GAD: Generalized Anxiety Disorder, GDA: general dietary advice, GFD: gluten-free diet, GI: gastrointestinal, GOS: galactooligosaccharides, GP: general practitioner, GSRS: Gastrointestinal Symptom Rating Scale, HADS: Hospital Anxiety and Depression Scale, H₂/CH₄: hydrogen/methane, IBS: irritable bowel syndrome, IDARS: IBS Diet Adherence Scale, ITT/mITT: intention-to-treat/modified intention-to-treat, LLD: low lactose diet, LNCT: Lactulose Nutrient Challenge Test, GC/LC/DI-MS: gas/liquid chromatography/direct infusion MS, LDA: linear discriminant analysis, LFD: low FODMAP diet, MD: Mediterranean diet, MFI-20: Multidimensional Fatigue Inventory, MID: minimal important difference, NICE: National Institute for Health and Care Excellence, NR: not reported, NS: not significant, NRS: Numeric Rating Scale, OR: odds ratio, OTU: operational taxonomic unit, PD: personalized diet, PHQ: Patient Health Questionnaire, PP: per-protocol, QoL: quality of life, RCT: randomized controlled trial, SCFA/BCFA: short/branched-chain fatty acids, SILFD: Structural Individual Low FODMAP Dietary Advice, SF-12: 12-item Short Form Survey, SF-NDI: Nepean Dyspepsia Index, SSRD: starch/sucrose-reduced diet, TBD: tritordeum-based diet, TDA: traditional dietary advice, TIBC: total iron-binding capacity, VC: volatile organic compound, VSI: Visceral Sensitivity Index, VAS: Visual Analog Scale, WHO: World Health Organization

Comparator	Study ID (author, year)	Design characteristics			Population							Intervention and comparator (I/C)				Extracted outcomes	Symptom scale used	QoL scale used	Key results
		Study design	Intervention duration (FODMAP restriction weeks)	Follow-up duration (weeks)	Country/setting	IBS diagnostic criteria	IBS subtype (included)	Total sample size (N)	Age (mean ± SD or median (IQR))	Sex (% female)	Baseline symptom severity	Intervention description (LFD)	Comparator description	Reintroduction phase included?	Concomitant treatments				
Traditional/NICE/TDA	Böhn et al., 2015 [8]	Multicenter, parallel, single-blind RCT	4	4	Sweden (gastroenterology outpatient clinics: Gothenburg and Stockholm)	Rome III	All subtypes (IBS-C, IBS-D, IBS-M/unsubtyped)	N = 75 randomized; 67 completed (LF n = 33, TDA n = 34)	43 ± 16 years (completers)	83.6% (56/67 completers)	LF: IBS-SSS 324 ± 69, TDA: IBS-SSS 302 ± 61	LFD: restricted fructans/GOS (wheat, rye, onion, legumes), lactose-containing products, excess fructose (apples, pears, honey), and polyols (apricots, artificially sweetened products). Patients given a pamphlet with food lists and alternatives	Traditional IBS dietary advice (NICE-based): regular meal pattern, avoid large/fatty/spicy meals, reduce caffeine, avoid beans/cabbage/onions. Focus on HOW and WHEN to eat rather than WHAT	No	IBS medications and antidepressants allowed (stable ≥1 month); probiotics allowed (unchanged intake); lactose-reduced diet allowed	IBS-SSS change from baseline; responder rate (IBS-SSS reduction ≥50), individual IBS-SSS subscales; stool consistency (BSF); stool frequency; dietary intake (4-day food diary); predictors of treatment response, BSF stool diary; 4-day food diary (FODMAP intake, nutrients); HADS; VSI; MFI-20; PHQ-15	IBS-SSS (0-500) + individual subscales	Not measured	Both groups showed significant symptom reduction (p < 0.0001). No statistically significant difference between groups in IBS-SSS change (LF: −77 ± 110 vs TDA: −65 ± 84; p = 0.62). Responder rates were similar: 50% (LF) vs 46% (TDA), p = 0.72. Responders to LF had lower baseline FODMAP intake. Low FODMAP group significantly reduced bowel frequency (p < 0.0001). Both groups reduced caloric intake despite not being advised to do so
	Chavan et al., 2025 [18]	Single-center, randomized, parallel-group RCT (3 arms)	8	8	India (Saiamrut Multispecialty Hospital, Satara, Maharashtra) - tertiary care	Rome IV	All subtypes (IBS-D 36%, IBS-C 30%, IBS-M 34%)	N = 150 randomized; 138 completed (LF n = 45, TDA n = 48, GFD n = 45)	LF: 39.8 ± 10.2, TDA: 42.1 ± 12.4, GFD: 41.5 ± 11.1	LF: 76%, TDA: 72%, GFD: 74%	LF: IBS-SSS 315.4 ± 55.2, TDA: IBS-SSS 308.1 ± 61.3, GFD: IBS-SSS 312.8 ± 58.7	LFD: restricted high FODMAP foods (wheat, onions, legumes, lactose, stone fruits) for 8 weeks. Based on Monash University food lists and recipes. 45-minute dietitian consultation	Arm 1: TDA. NICE guidelines: small regular meals, avoid large/fatty meals, reduce caffeine/alcohol/fizzy drinks, adjust fiber. Arm 2: GFD: exclude all gluten sources (wheat, barley, rye); other FODMAPs (onions, garlic, pulses) permitted	No	Not specified	IBS-SSS change at 8 weeks; IBS-QoL change at 8 weeks; dietary adherence (Likert scale); global symptom relief (binary adequate relief); adherence score (Likert 0–10); adequate relief (yes/no); food-related anxiety	IBS-SSS (0-500), IBS-QoL (0-100)	IBS-QoL (0–100)	LF produced the greatest reduction in IBS-SSS (−145.2 ± 42.1) vs GFD (−92.4 ± 38.5) vs TDA (-76.8 ± 35.2), p < 0.001. IBS-QoL improved most with LF (+18.4 ± 9.5) vs GFD (+13.5 ± 8.8) vs TDA (+10.2 ± 8.2), p = 0.02. Adequate relief was highest with LF (76%) vs GFD (64%) vs TDA (54%), p = 0.04. However, TDA had the highest adherence (88%) vs GFD (70%) vs LF (62%), p = 0.01. LF was also associated with the highest food-related anxiety
	Eswaran et al., 2016 [19]	Single-center, parallel, superiority RCT	4	4	USA (University of Michigan) - gastroenterology and primary care clinics	Rome III	IBS-D only	N = 92 (LF n = 47, mNICE n = 45) randomized; 84 completed (LF n = 45, mNICE n = 39)	LF: 41.6 ± 14.70, mNICE: 43.8 ± 15.2	LF: 66%; mNICE: 76.2%	LF: pain 5.10 ± 1.5, bloating 4.87 ± 1.83, BSFS 5.21 ± 0.60, mNICE: pain 5.06 ± 1.34, bloating 5.01 ± 2.07, BSFS 5.25 ± 0.70	LFD: based on Monash University materials; restricted fructose, lactose, fructans, GOS, polyols. Counseled by a trained research dietitian. 3-day food diary monitoring	Modified NICE (mNICE) guidelines: small frequent meals, avoid trigger foods, avoid excess alcohol/caffeine. FODMAPs NOT specifically excluded	No	Stable antidepressants allowed; no probiotics, antibiotics, or narcotics	Adequate relief of IBS-D symptoms ≥50% of weeks 3-4 (primary); FDA composite endpoint (≥30% pain reduction + BSFS decrease ≥1); pain responders (≥30% reduction); stool consistency responders (BSFS decrease ≥1); daily: abdominal pain score, bloating score, urgency score, BSFS, stool frequency	11-point NRS (pain, bloating, urgency); BSFS; adequate relief (weekly binary)	NR in this paper	Primary endpoint (adequate relief) NS different: LF 52% vs mNICE 41% (p = 0.31). FDA composite also NS: LF 27% vs mNICE 13% (p = 0.13). However, LF was significantly superior for pain responders (51% vs 23%, p = 0.008). Mean improvements in abdominal pain, bloating, stool consistency, frequency, and urgency were all significantly greater in LF vs mNICE from week 1 through week 4 (all p < 0.05). Caloric intake decreased in both groups. Dietary adherence confirmed by food diaries
	Goyal et al., 2021 [20]	Prospective, randomized, single-blind trial	4 (phase I strict LFD)	16 (4-week restriction + 12-week reintroduction)	India/tertiary care (Dayanand Medical College, Punjab) - IBS-D patients	Rome IV (IBS-D only)	IBS-D only	Screened N = 166; randomized N = 101 into LFD (n = 52) and TDA (n = 49)	41.9 ± 17.1 years	42% (58% male)	IBS-SSS: 261.6 ± 133.3 (LFD), 268.1 ± 121.3 (TDA); IBS-QOL: 57.2 ± 12.3 (LFD), 55.1 ± 12.9 (TDA)	Strict LFD (<0.5 g FODMAPs/serving) for 4 weeks (phase I), then systematic FODMAP reintroduction ('modified LFD') for 12 weeks (phase II). Dietitian-delivered, written instructions + food diary	TDA: small frequent meals, adequate fluids, reduce alcohol/caffeine/fat/spicy food, limit fiber. Continued for 16 weeks	Yes (phase II: systematic FODMAP rechallenge 4-16 weeks)	IBS medications permitted (antispasmodics, anti-anxiety, antidepressants, antidiarrheals, etc.); assessed and tracked	IBS-SSS total and subscores (pain severity/frequency, abdominal distension, bowel habits dissatisfaction, life interference); IBS-QOL; BSFS; stool frequency; proportion of responders (IBS-SSS reduction ≥50); adequate symptom relief; macronutrient and FODMAP intake; dietary compliance; medication use; adverse events; predictors of response	Symptom scale: IBS-SSS (0-500) + individual subscores; BSFS; stool frequency diary	QoL scale: IBS-QOL (0-100)	This Indian RCT (n = 101 IBS-D) compared a strict 4-week LFD followed by 12-week FODMAP reintroduction versus TDA. At 4 weeks, IBS-SSS was significantly lower in the LFD group (126.4 ± 75.7) than in the TDA group (186.7 ± 98.1; p = 0.002), and IBS-QOL significantly improved (64.3 ± 13.2 vs. 60.4 ± 11.1; p = 0.048). By ITT, responders at 4 weeks were 62.7% (LFD) vs. 40.8% (TDA; p = 0.045). At 16 weeks after FODMAP reintroduction, LFD remained superior: IBS-SSS 151.7 ± 91.2 vs. 202.4 ± 114.7 (p = 0.014), responders 52.9% vs. 30.6% (p = 0.027). Stool frequency and consistency improved significantly only in the LFD group. IBS-QOL was significantly higher in LFD at 16 weeks (71.3 ± 14.4 vs. 64.5 ± 14.1; p = 0.016). Nutritional reductions in energy, carbohydrate, fat, and fiber at 4 weeks recovered at 16 weeks after reintroduction. Medication use was significantly lower in LFD at 16 weeks (p = 0.028)
	Kasti et al., 2025 [21]	Randomized, controlled, parallel-group study	8-14 (2-6 weeks restrictive + 6-8 weeks reintroduction)	~24 (6-month follow-up)	Greece (Attikon University General Hospital, Athens) - tertiary gastroenterology unit	Rome IV	Non-constipated IBS (IBS-D, IBS-M); IBS-C excluded	N = 108 randomized; 103 at 1st FU; 92 completed study (MED-LFD n = 54, NICE n = 54)	MED-LFD: 39 ± 11, NICE: 41 ± 12	MED-LFD: 79.6%; NICE: 75.9%	MED-LFD: IBS-SSS 308 ± 79, NICE: IBS-SSS 321 ± 70	Mediterranean LFD (MED-LFD): 3-phase diet. Phase 1 (2-6 weeks): FODMAP intake 0.77-1.91 g/day; enriched with Mediterranean elements (olive oil, fish, vegetables, fruits). Phase 2 (6-8 weeks): gradual FODMAP reintroduction. Phase 3: long-term PD. Dietitians certified by Monash University. Weekly phone monitoring	NICE dietary guidelines for IBS: weekly meal plan for 4 weeks covering regular meals, fiber adjustment, caffeine/alcohol/fatty food reduction. Up to 3 consultations over 6 months	Yes (MED-LFD group completed full 3-phase protocol including reintroduction)	Rescue medications permitted: paracetamol, hyoscine butyl bromide, antispasmodics, loperamide. Routine IBS medications not permitted during study	IBS-SSS change from baseline (primary); responder rate (IBS-SSS reduction ≥50 points) at 1st and 2nd follow-up, GSRS-IBS (symptom burden); IBS-QoL; SF-12; HADS (anxiety/depression); BSFS; adequate relief; adherence; SCFA/BCFA stool analysis (exploratory), HADS (anxiety/depression); BSFS; FODMAP Adherence Report Scale (FARS); stool SCFA/BCFA (GC/MS exploratory subgroup n = 40)	IBS-SSS (0-500), GSRS-IBS	IBS-QoL (0-100), SF-12	At first follow-up, IBS-SSS was significantly lower in MED-LFD (159 ± 80) than in NICE (253 ± 94), p < 0.001. Responder rate: MED-LFD 84.6% vs NICE 60.8% (p = 0.007). At 6 months: MED-LFD 79.1% vs NICE 52.3% (p = 0.006). IBS-QoL improved more in MED-LFD (+24.2 vs +8.9 points, p < 0.001). Adequate relief at 1st FU: 87% vs 64.8% (p = 0.007). GSRS-IBS, SF-12, HADS anxiety, and depression were all significantly superior in MED-LFD. Adherence was higher in MED-LFD (75% vs 41%). Stool SCFAs and BCFAs were significantly reduced in both groups. MED-LFD was the strongest independent predictor of being a responder (OR = 6.66 at 1st FU)
	Patcharatrakul et al., 2019 [22]	Prospective, randomized, single-blind, parallel-group controlled trial	4	4 (no post-intervention follow-up)	Thailand/outpatient gastroenterology (King Chulalongkorn Memorial Hospital, Bangkok)	Rome III	IBS-C and IBS non-constipation (IBS-D, IBS-M)	66 randomized; 62 completed (SILFD n = 30, BRD n = 32)	Mean 51 ± 14 years	76%	Global IBS symptom score (VAS 0-100): SILFD 61.2 ± 21.0; BRD 56.3 ± 17.8; high FODMAP items/week: 16 in both groups	SILFD: personalized identification of high FODMAP items from an individual's 7-day food diary, then replacement with low FODMAP items (using a country-specific pamphlet and menu). Up to a 30-minute session with a gastroenterologist. No formal dietitian involvement	Brief advice on a commonly recommended diet (BRD): 5-min advice to reduce traditionally gas/pain-triggering foods (fruits, vegetables, nuts, beans, garlic) and avoid large meals. Term "FODMAP" not used	No	Stable IBS medications required ≥4 weeks before and during study; antibiotics/probiotics excluded	Primary: responder rate (≥30% decrease in average daily worst abdominal pain/discomfort at week 4); global IBS symptom severity (VAS 0–100); individual symptom VAS scores (abdominal pain, discomfort, bloating, belching, stool urgency); stool frequency (IBS-C vs non-C separately); number of high FODMAP food items/week (dietary compliance by food diary); postprandial breath hydrogen (H2) and methane (CH4) AUC and peak over 4-h period; HADS (anxiety and depression scores)	Symptom scale: Global IBS symptom severity VAS (0-100); individual symptom VAS scores (abdominal pain, discomfort, bloating, belching, urgency); responder criterion ≥30% reduction in worst daily abdominal pain	Not measured	In this Thai single-blind RCT (n = 62), 60% of SILFD patients met the responder criterion (≥30% reduction in worst daily abdominal pain/discomfort) vs. 28% in the BRD group (p = 0.001). Global IBS symptom severity (VAS 0-100) improved significantly in SILFD (61.2 → 38.5; p < 0.001) but not in BRD (56.3 → 53.5; p > 0.05), with a significant between-group difference at the end of the study (p = 0.006). Abdominal pain, discomfort, and bloating decreased significantly from baseline only in the SILFD group. High FODMAP item consumption significantly decreased in SILFD (16.0 → 9.6 items/week; p < 0.001) but not in BRD (16.2 → 15.4; p > 0.05). Postprandial breath H2 AUC was significantly lower after SILFD than after BRD (795 vs. 2520 ppm-min; p < 0.001), confirming reduced intestinal fermentation. In the IBS-constipation subgroup, SILFD was most superior (73% vs. 28% responder rate; p < 0.05). Breath CH4 trended lower after SILFD but did not reach significance
	Rej et al., 2022 [23]	RCT (parallel, 3-arm: LFD vs TDA vs GFD)	4	4 (no post-diet follow-up)	UK/secondary care (Sheffield Teaching Hospitals NHS Foundation Trust)	Rome IV	IBS-D, IBS-M (non-constipated only)	99 (33 per arm)	37 ± 13 years	71%	IBS-SSS: 301 ± 89 overall; 9% mild, 47% moderate, 45% severe	LFD for 4 weeks (<12 g/day FODMAPs target). Standardized 45-60 minutes face-to-face or virtual dietitian session with written dietary information sheets	TDA: regular meals, adequate hydration, reduce alcohol/caffeine/fizzy drinks/fatty/spicy/processed foods, reduce fresh fruit to max 3/day, reduce gas-producing foods (beans, bread, sweeteners), address food intolerances, consistent with NICE/BDA guidelines. Also: GFD as third arm (gluten cross-contamination allowed)	No	Not restricted	IBS-SSS total and subscores (abdominal pain severity/frequency, abdominal distension, bowel habit dissatisfaction, life interference); IBS-QOL (34-item); HADS (anxiety, depression); PHQ-12 (somatic symptoms); acceptability of dietary restriction questionnaire; food-related QOL; nutritional intake (CNAQ: macro/micronutrients, FODMAP components); stool dysbiosis index (GA-map); clinical response rate (≥50-point IBS-SSS reduction); responder predictors	IBS Symptom Severity Score (IBS-SSS): 5-item, 0-500, primary endpoint ≥50-point reduction	IBS-QOL (34-item, 0-100, higher = better QoL); also HADS (anxiety/depression), PHQ-12 (somatic symptoms), food-related QoL questionnaire, acceptability of dietary restriction questionnaire	This UK 3-arm RCT (n = 99) compared LFD, TDA, and GFD in non-constipated IBS-D/M. The primary endpoint (≥50-point IBS-SSS reduction at 4 weeks) was met by 42% on TDA, 55% on LFD, and 58% on GFD, with no significant difference (p = 0.43). Responders in all groups had significant within-group improvements in all IBS-SSS subscores, with no difference across diets. TDA was significantly cheaper (p < 0.01), less time-consuming to shop for (p < 0.01), and easier to follow when eating out (p = 0.03) than GFD and LFD; TDA and GFD were easier to incorporate into daily life than LFD (p = 0.02). LFD reduced FODMAP intake the most (27.7 to 7.6 g/day), followed by TDA (24.9 to 15.2 g/day) and GFD (27.4 to 22.4 g/day; p < 0.01). Stool dysbiosis index changes were similar across groups (p = 0.99). LFD was associated with greater improvement in depression and dysphoria on IBS-QOL vs TDA. No baseline clinical or microbial factor predicted dietary response. The authors recommend TDA as first-choice dietary therapy in non-constipated IBS
	Zahedi et al., 2018 [24]	Randomized, controlled, single-blind, parallel-group trial	6	6 (no post-diet follow-up)	Iran/tertiary care (Afzalipour Hospital, Kerman University of Medical Sciences)	Rome III	IBS-D only	101 randomized; 101 completed (50 LFD, 51 GDA)	LFD: 37.60 ± 11.09 years; GDA: 37.43 ± 13.27 years	LFD: 48% female; GDA: 53% female	IBS-SSS: LFD 263.75 ± 91.25; GDA 252.5 ± 85.51; Stool consistency (Bristol): LFD 5.92 ± 0.45; GDA 5.67 ± 0.61	LFD (<0.5 g FODMAPs per meal) for 6 weeks. Individual 45-min session with a specialized dietitian. Written pamphlet with suitable/unsuitable foods, shopping guide, strategies for eating out, cooking instructions. Culturally adapted for Iranian foods	GDA based on British Dietetic Association recommendations: limit caffeine, alcohol, spicy, fatty, and carbonated foods; eat small, frequent meals; eat slowly; avoid polyol-containing chewing gum and sweeteners	No	Antidepressants at stable dose permitted; GI motility drugs excluded; probiotics excluded	IBS-SSS total and subscores at weeks 0, 3, 6 (abdominal pain intensity/frequency, abdominal distension, intestinal transit dissatisfaction, daily life interference); IBS-QOL (0-100, higher = poorer QoL); HADS (anxiety, depression); BSFS (consistency); stool frequency; 3-day food diary (energy, macronutrients, fructose, lactose, polyols)	IBS-SSS (5-item VAS, 0-500); BSFS (stool consistency); stool frequency diary	IBS-QOL (0-100)	In this Iranian single-blind RCT (n = 101 IBS-D), both the LFD and GDA groups showed significant reductions in IBS-SSS at 6 weeks vs. baseline (p < 0.001 in both groups). However, the LFD was significantly superior, with a greater reduction in total IBS-SSS (LFD: 263.75 → 108 ± 63.82 vs. GDA: 252.5 → 149.75 ± 51.39; p < 0.001 between groups at week 6). All five IBS-SSS subscores improved significantly more in the LFD group (abdominal pain intensity p = 0.001, pain frequency p = 0.017, abdominal distension p < 0.001, intestinal transit dissatisfaction p = 0.001, life interference p = 0.005). Stool frequency (1.91 vs. 2.6 per day; p < 0.001) and consistency (Bristol 4.3 vs. 4.61; p = 0.003) also improved significantly more with LFD. IBS-QOL improved significantly within both groups (p < 0.001 for LFD; p = 0.001 for GDA), but there was no significant difference between groups at the end of the study (p = 0.332). The authors conclude that LFD has greater benefits for GI symptoms than GDA in IBS-D and recommend it as a first-line treatment in combination with other approaches
	Zhang et al., 2021 [25]	Parallel-group, RCT (single-center)	3	3 (no post-diet follow-up)	China/single-center academic hospital (Sir Run Run Shaw Hospital, Zhejiang University School of Medicine, Hangzhou)	Rome III	IBS-D only	108 randomized; 100 completed (51 LFD, 49 TDA)	LFD: 42.4 ± 10.7 years; TDA: 44.6 ± 14.6 years	LFD: 46.3% female; TDA: 48.1% female	IBS-SSS: LFD 244.6 ± 76.3; TDA 231.2 ± 69.4; IBS-QOL: LFD 41.4 ± 24.6; TDA 33.3 ± 22.5	LFD for 3 weeks. Patients instructed to avoid high FODMAP foods (per Monash University app). Minimum 20-min face-to-face dietitian consultation + 3-week meal plan. Weekly telephone compliance monitoring. Dietitians trained at the University of Michigan	TDA based on modified NICE guidelines: reduce fatty/spicy food, coffee, alcohol; eat regularly 3 times/day; not too much/too little; eat in peace with thorough chewing. FODMAPs not specifically excluded	No	Antidepressants at stable dose ≥6 weeks permitted; antibiotics, probiotics, dietary supplements excluded	IBS-SSS (primary: ≥50-point reduction at 3 weeks); IBS-QOL; GAD-7; PHQ-9; daily symptom diary (abdominal pain NRS 0-10, stool consistency BSFS, stool frequency, abdominal bloating, excessive wind, urgency, incomplete defecation, overall discomfort 4-pt scale); fecal SCFAs (acetic, propionic, n-butyric, i-butyric, valeric, i-valeric acids; fermentation index A and B); fecal microbiota 16S rRNA (α/β-diversity, LEfSe); predictors of LFD response	BS-SSS (primary); daily symptom diary: abdominal pain NRS (0-10), stool frequency, stool consistency (BSFS), abdominal bloating (4-pt scale), excessive wind, urgency, incomplete defecation, overall discomfort	IBS-QOL; GAD-7 (General Anxiety Disorder 7); PHQ-9 - depression	In this Chinese parallel RCT (n = 100 completers, IBS-D), both LFD and TDA improved IBS-D symptoms over 3 weeks with similar primary endpoint rates: 59% (LFD, PP) vs. 53% (TDA, PP; p = 0.56; ITT: 55.6% vs. 48.1%; p = 0.44). However, LFD achieved earlier symptomatic improvements vs. TDA: stool frequency (p = 0.005) and excessive wind (p = 0.011) improved significantly more in LFD at week 1. At week 3, both groups showed comparable improvement in most symptoms. Baseline FODMAP intake was already relatively low in this population (LFD: 6.32 g/day; TDA: 5.77 g/day), which may explain the modest LFD advantage. LFD reduced FODMAP intake to 1.49 g/day (p < 0.001) vs. no change in TDA. LFD reduced carbohydrate-fermenting bacteria (*Bifidobacterium*, *Fusobacterium*) and n-butyric acid, and increased microbial α-diversity. Higher baseline IBS-SSS (p = 0.01) and higher saccharolytic fermentation index A (p = 0.05) predicted a favorable LFD response. Conversely, a high fermentation index predicted a worse TDA response. A low fermentation index B was independently associated with response to both diets
Habitual/normal diet/no intervention	Algera et al., 2022 [26]	Randomized, double-blind crossover trial	1 (7 days)	5 (incl. 14-day washout)	Sweden/secondary care (University Hospital)	Rome IV	IBS-C, IBS-D, IBS-M, IBS-U	29	31 ± 10 years	90%	IBS-SSS: 316 ± 75 (moderate-severe); IBS-SSS screening cutoff ≥175	LFD: 4 g FODMAPs/day (7 days). Meal boxes + snacks provided by hospital kitchen. NICE behavioral advice also given	Moderate FODMAP diet: 23 g FODMAPs/day (7 days). Same meal structure as intervention	No	Not restricted; NICE lifestyle advice (limit alcohol, caffeine, fatty/spicy foods) given to both groups	IBS-SSS total and subscores (abdominal pain intensity/frequency, bloating, bowel habits dissatisfaction, daily life interference); BSFS (consistency, frequency); HADS (anxiety, depression); VSI (GI-specific anxiety); PHQ-12 (somatic symptoms); LNCT GI symptoms and exhaled H2/CH4; clinical response rate; FODMAP sensitivity rate	Symptom scale: IBS-SSS (0-500) + individual subscores; BSFS (consistency and frequency)	QoL scale: not measured (HADS and VSI assessed, but no formal QoL scale	In this 7-day double-blind crossover trial (n = 29), IBS patients on a LFD (4 g/day) showed significantly reduced overall GI symptom severity vs. a moderate FODMAP diet (23 g/day). IBS-SSS decreased significantly after low FODMAP (mean change: −57 ± 108) but not after moderate FODMAP (−10 ± 72; p = 0.04 between interventions). A third (34%) clinically responded to the LFD (IBS-SSS reduction ≥50 points), and higher baseline IBS-SSS independently predicted response (OR 1.04 per point, p = 0.02). Stool consistency became firmer (p = 0.03), and frequency decreased (p < 0.01) on the LFD. FODMAP sensitivity (22%) was modestly associated with worse GI symptoms during LNCT, but no independent predictor was identified. Psychological and non-GI somatic symptoms did not differ between diets
	Harvie et al., 2017 [27]	RCT (parallel design, waitlist comparator)	12 (3 months education + implementation)	24 (6 months; reintroduction at 3 months for group I)	New Zealand/mixed settings (GI outpatient, GP, community)	Rome III	IBS-D, IBS-C, IBS-M	50	Group I: 43.3 ± 13.8 years; group II: 40.6 ± 13.3 years	Group I: 73%; group II: 96%	IBS-SSS: group I: 272 ± 60; group II: 254 ± 80	Dietitian-delivered low FODMAP education (1-hour initial session + follow-up). Group I started immediately at baseline; Group II started at 3 months. Patients purchased/prepared their own food. Written resources from Monash University	Waitlist control (group II received no dietary education during the first 3 months)	Yes (group I started FODMAP reintroduction at 3 months, guided by a dietitian)	NR (antibiotics, IBD, celiac disease exclusion criteria)	IBS-SSS total and subscores (bloating severity, pain severity, pain frequency); IBS-QOL total and subscales; FODMAP-specific FFQ; energy, fiber, and calcium intake; gut microbiome (16S rRNA, Shannon diversity, OTUs); bowel motion frequency; pain frequency	Symptom scale: IBS-SSS (0-500) + individual subscores	QoL scale: IBS-QOL (0-100, higher = better)	In this 6-month RCT (n = 50), group I received immediate LFD education and started FODMAP reintroduction at 3 months; group II was a waitlist control and received education at 3 months. At 3 months, group I showed a significantly larger IBS-SSS reduction vs. group II (−144.5 ± 89.0 vs. −38.7 ± 74.8; p < 0.01), with IBS-SSS of 128.8 ± 82.5 vs. 203.6 ± 70.1. IBS-QOL improved clinically and statistically in group I (68.5 → 83.0) vs. no change in group II (72.9 → 73.3; p < 0.01). Pain frequency reduced significantly more in group I (p < 0.01). At 6 months, symptom reduction was sustained in Group I (IBS-SSS 160 ± 102) and replicated in group II after starting the diet (IBS-SSS 124 ± 76). During the strict phase, fiber intake fell (33 → 21 g/day; p < 0.01) but recovered after FODMAP reintroduction (27 g/day). Gut microbiome diversity did not change significantly. The diet was most effective for IBS-D patients
	Nilholm et al., 2019 [28]	Randomized, open clinical trial	2	2	Sweden/secondary and tertiary care (Skåne University Hospital, Malmö)	Rome IV	IBS-C, IBS-D, IBS-M, IBS-U	105 randomized; 98 completed	46.06 ± 13.11 years	78.1%	IBS-SSS median 310 (248-353); 52.4% severe disease (≥300); IBS-SSS ≥175 required for inclusion	SSRD for 2 weeks: avoidance of all sucrose-containing foods; reduced cereal intake (≤1 serving/day); increased meat, fish, vegetables, fruits, eggs, dairy. Fiber-rich alternatives preferred. Added fat/protein to delay GI transit. Nuts instead of sweets. Written and oral guidance by study staff	Control: continued ordinary eating habits during 2-week observational period. Received dietary information after study completion	No	Not restricted; patients on vegan, gluten-free, LCHF, or LFD at baseline were excluded	IBS-SSS total and subscores; VAS-IBS (abdominal pain, diarrhea, constipation, bloating/flatulence, vomiting/nausea, psychological well-being, daily life interference); 4-day food diaries (meal frequency, food categories); Blood samples (iron, ferritin, TIBC, folic acid, cobalamin, 25-OH vitamin D, CRP); Responder rate (IBS-SSS reduction ≥50 pts)	Symptom Scale: IBS-SSS (0-500) + individual subscores; VAS-IBS (abdominal pain, diarrhea, constipation, bloating/flatulence, nausea, psychological well-being, daily life interference)	Not measured	In this Swedish 2-week open RCT (n = 105 IBS), an SSRD significantly improved multiple GI symptoms vs. controls continuing habitual diet. The intervention group showed significantly greater reductions in total IBS-SSS (p < 0.001), abdominal pain (p = 0.001), diarrhea (p = 0.002), bloating/flatulence (p = 0.005), psychological well-being (p = 0.048), and daily life interference (p < 0.001) vs. controls. 66.3% of intervention patients were responders (IBS-SSS reduction ≥50 pts) vs. 20% of controls (p < 0.001); 31.1% vs. 0% met the 50% reduction threshold. Patients with irregular eating habits had higher IBS-SSS (p = 0.029). Decreased intake of cereals and sweets correlated with lower scores. The control group showed no significant changes. 77% of patients had vitamin D below reference values. Symptoms typically improved within 2-3 days of starting SSRD
	Staudacher et al., 2011 [12]	RCT (parallel)	4	4	UK/secondary care (Guy's and St Thomas' NHS Foundation Trust, London)	Rome III (clinical IBS with bloating and/or diarrhea)	IBS with predominant bloating and/or diarrhea (IBS-D; IBS-C excluded)	41 randomized; 35 ITT (19 LFD, 16 control per group)	NR	NR	NR (GSRS and global symptom question used; IBS-SSS NR)	LFD for 4 weeks: dietitian-delivered guidance identifying and restricting high FODMAP foods using standard UK RD approach	Habitual diet (control): no dietary change advised. Same number of follow-up contacts as LFD group	No	Exclusion: other GI disorders, previous GI surgery, antibiotics/probiotics/prebiotics within 4 weeks	GSRS incidence and severity by symptom (bloating, borborygmi, urgency, overall symptoms); global symptom question (adequate symptom control); stool frequency and consistency (Bristol Stool Chart); fecal pH; proportion with symptom improvement per domain	Symptom scale: GSRS - incidence and severity by domain; global symptom question (adequate symptom control); Bristol Stool Chart	Not measured	In this first RCT of an LFD in IBS (n = 41 randomized, 35 ITT), patients on the LFD for 4 weeks showed significantly greater symptom improvement vs. habitual diet controls. More LFD patients improved in bloating (74% vs. 32%; p = 0.007), borborygmi (68% vs. 36%; p = 0.041), urgency (53% vs. 23%; p = 0.047), and overall symptoms (79% vs. 36%; p = 0.006). Adequate symptom control was reported by 68% (LFD) vs. 23% (control; p = 0.003). PP: lower bloating incidence (3.6 vs. 5.9 days; p = 0.007) and severity (0.8 vs. 1.5; p = 0.013) and lower overall symptom incidence/severity on LFD. Stool frequency was lower in LFD (9.3 vs. 14.2 stools/week; p = 0.025). Trend toward more normal stool consistency in LFD (33% vs. 16%; p = 0.059). No significant difference in patient-reported diarrhea or fecal pH between groups. This study established the foundational evidence for the LFD in IBS
Higher FODMAP/ typical diet	McIntosh et al., 2017 [29]	Prospective, randomized, single-blind, parallel-group controlled trial	3 (21 days)	3 (21 days; no post-diet follow-up)	Canada/academic teaching hospital outpatient (Kingston General Hospital, Ontario)	Rome III	IBS-C, IBS-D, IBS-M, IBS-U	40 randomized; 37 completed (19 low FODMAP, 18 high FODMAP)	Low FODMAP: 50.28 years (range 26-77); high FODMAP: 51.47 years (range 24-83)	Low FODMAP: 83%; high FODMAP: 89%	IBS-SSS: Low FODMAP 289.6 ± 81.3; high FODMAP 271 ± 80	LFD for 3 weeks. Dietitian-provided dietary booklet with sample menus specifying low FODMAP foods. Individual 30-60 minute education session with GI dietitian. Diet diary kept	High FODMAP diet for 3 weeks. Same dietitian structure; booklet specified high FODMAP foods. Both groups blinded to which diet was "better"	No	Antibiotics, stool bulking agents, narcotic analgesics, lactulose prohibited. No probiotics taken	IBS-SSS total and subscores (abdominal pain intensity/frequency, abdominal distension, bowel habit dissatisfaction, life interference); proportion of responders (IBS-SSS reduction ≥50); lactulose breath test (H2 AUC); urinary metabolomics (DI-MS, GC-MS, LC-MS: histamine, p-hydroxybenzoic acid, azelaic acid); fecal microbiota 16S rRNA profiling (α- and β-diversity, genus-level changes); FODMAP dietary diary score (compliance)	Symptom scale: IBS-SSS (0-500) + individual subscores; FODMAP diet diary compliance score	Not measured	In this single-blind RCT (n = 37 completers), IBS patients randomized to a LFD for 3 weeks showed a significant 28% reduction in IBS-SSS (289.6 → 208.0; p < 0.001), whereas those on a high FODMAP diet reported a non-significant 7% increase (271 → 290; p = 0.57). The proportion of responders (IBS-SSS reduction ≥50) was significantly greater in the low FODMAP group (72%; 13/18) vs. high FODMAP (21%; 4/19; p = 0.009). Abdominal pain intensity decreased by 52% in the low FODMAP group (p < 0.01), and pain days increased in the high FODMAP group (p < 0.05). FODMAP diet diary scores significantly correlated with IBS-SSS (R² = 0.25; p = 0.002). Urinary metabolomics revealed an 8-fold reduction in histamine in the low FODMAP group (p < 0.05), a marker of immune/mast cell activation. The LFD increased Actinobacteria richness/diversity; the high FODMAP diet reduced species richness and decreased butyrate producers (Roseburia, Ruminococcus). *Bifidobacterium* increased in the high FODMAP group. Multiple microbiome-symptom and microbiome-metabolite correlations were identified
	Tuck et al., 2019 [30]	Randomized crossover trial (longitudinal, single-blind)	3	11	Canada (Queen's University, Kingston, ON)/academic hospital	Rome IV	IBS-D (n = 7), IBS-C (n = 2), IBS-M (n = 1)	N = 10 (interim analysis; all female)	NR (adults)	100% female	IBS-SSS baseline: 273.9 ± 78.7; SF-NDI QOL: 26.7 ± 6.3; Total FODMAP intake 9.1 ± 6.3 g/day	LFD for 3 weeks. Detailed dietary instruction by a specialized dietitian at the start. Advised that either diet may have therapeutic potential. 7-day food diary, IBS-SSS, SF-NDI QOL, and fasting urine samples collected at the end of each period	High FODMAP diet for 3 weeks (same dietitian instruction format). Two non-intervention periods: 1-week baseline and 3-week run-in prior to dietary interventions	No	NR	IBS-SSS; SF-NDI QOL; total FODMAP intake (g/day); 7-day food diary; urinary metabolomics (117 metabolites by LC-MS); urine phosphatidylcholines, sphingomyelins, acylcarnitines	IBS-SSS (0-500)	SF-NDI (QOL)	This interim analysis of a longitudinal crossover RCT (n = 10, all female) included 1-week baseline, 3-week run-in, and two 3-week dietary phases (low and high FODMAP, randomized order). Symptoms were stable between the two non-intervention periods (p = NS). The LFD significantly reduced IBS-SSS vs. baseline (IBS-SSS: 190.2 ± 60.4 vs. 273.9 ± 78.7; p = 0.00). LFD showed a trend toward lower IBS-SSS (270.4 ± 103.9 vs. 190.2 ± 60.4; p = 0.07). SF-NDI QOL improved significantly with low FODMAP (19.5 ± 6.3) vs. baseline (26.7 ± 6.3; p = 0.04). Total FODMAP intake confirmed dietary compliance (low FODMAP: 2.8 ± 1.4 g/day vs. high FODMAP: 8.3 ± 5.2 g/day; p = 0.01). Urinary metabolomics: LFD significantly altered the metabolome vs. baseline, primarily driven by phosphatidylcholines (PC40:2AA, PC36:0AA, LYSOC18:2). A metabolomic shift was also observed between low and high FODMAP diets, mainly involving sphingomyelins (SM 20:2), phosphatidylcholines (LYSOC20:4, PC40:6AE), and acylcarnitines (C18:1OH). A larger sample is needed to characterize the full metabolomic profile
Other active dietary interventions (non-NICE)	Caponio et al., 2022 [31]	RCT (parallel, 2 arms)	12	12 (no post-diet follow-up)	Italy/outpatient gastroenterology (National Institute of Gastroenterology "S. de Bellis," Castellana Grotte)	Rome IV	IBS-D only	38 (20 TBD, 18 LFD)	45.7 ± 10.2 (TBD); 42.9 ± 10.3 (LFD)	TBD: 85% (17F/3M); LFD: 78% (14F/4M)	IBS-SSS: 284.3 ± 92.8 (TBD); 261.7 ± 67.0 (LFD), both moderate severity	LFD for 12 weeks, supervised by trained nutritionists. Controlled diet limiting fermentable oligosaccharides, disaccharides, monosaccharides, and polyols. Booklet with allowed/avoided foods provided. Food diary kept throughout. Daily macronutrients: 50% carbohydrates, 30% lipids, 20% protein	TBD for 12 weeks - diet using all food items (bread, pasta, biscuits) made exclusively from tritordeum flour (a cross between durum wheat and wild barley). Tritordeum products freely provided monthly. Same macronutrient targets	No	No antibiotic, probiotic, SSRI, antidepressant, or IBS-specific drugs within 2 weeks prior; lactose-reduced diet allowed if maintained constant	IBS-SSS total score; VOCs - 126 VOCs by HS-SPME GC-MS (grouped: alcohols, esters, aldehydes, phenols, ketones, organic acids, terpenes, hydrocarbons, indoles, furans, lactones); SCFAs (acetic, propanoic, butanoic, isobutyric, isovaleric acid) by GC-MS; Spearman correlations between VOCs and IBS-SSS; nutritional intake (food diary + software); IDARS adherence scale	Symptom scale: IBS-SSS (0-500) total score only	QoL scale: not measured (fecal metabolomics and SCFAs were the primary secondary outcomes)	This 12-week parallel RCT (n = 38 IBS-D) compared the LFD and a TBD on the fecal metabolome. Both diets significantly reduced IBS-SSS total scores from moderate to mild severity (TBD: 284.3 → 164.1, −42.8%, p < 0.0001; LFD: 261.7 → 113.8, −56.5%, p < 0.0001), with a non-significant trend favoring LFD (p = 0.10). Fecal VOC profiles did not differ significantly between groups at baseline. After 12 weeks, LFD was characterized by significantly higher aldehydes (3-methyl-butanal, benzaldehyde), terpenes (alpha-muurolene, gamma-terpinene), esters (pentanoic acid ethyl ester, hexanoic acid butyl ester), and phenethyl alcohol vs. TBD (p < 0.05). TBD had higher alpha-farnesene (p < 0.05). Within groups, LFD significantly decreased ethanol and nonanal (both pro-inflammatory markers; p < 0.05) and increased alpha-muurolene. TBD significantly increased decanoic acid (anti-inflammatory) (p < 0.05). SCFA analysis showed significant decreases in acetic acid (LFD: −57%, p = 0.030; TBD: −67%, p = 0.009) and propanoic acid (TBD: −38%, p = 0.031; LFD: −33%, NS) after both diets, consistent with improved IBS
	Jeitler et al., 2021 [32]	Open-label multicenter RCT (non-inferiority, exploratory)	12	24 (6-month total; follow-up at 1, 3, 6 months)	Germany (Immanuel Hospital Berlin and Evang. Kliniken Essen-Mitte, outpatient)	German S3 IBS guideline (close to Rome III/IV)	All subtypes	69	Conv: 41.8 ± 14.4 years; Ayurveda: 50.8 ± 12.0 years	75.4% overall (Conv 70.6%; Ayurveda 80%)	IBS-SSS mean: Conv 279 ± 78.2; Ayurveda 272.7 ± 77.1	Conventional nutritional therapy (German Nutrition Society guidelines) including LFD: 3 sessions (45-minute baseline + two 30-min follow-ups at 3 and 8 weeks). Brochure with balanced diet principles + low FODMAP guidance. Avoid fructans/GOS (wheat, rye, onions, legumes), lactose, excess fructose (apples, mangoes, honey), sorbitol/mannitol/xylitol (apricots, peaches). Low FODMAP recipes and food lists provided. After 12-week elimination: FODMAP reintroduction by food group (2-3 days each)	Ayurvedic nutritional therapy: Individualized dietary recommendations based on Ayurvedic "digestive fire" (agni) concept. Warm food, regular meal timings, light-but-nourishing foods. Customized to individual symptoms/constitution. 3 sessions with experienced Ayurvedic practitioners (>500 h training, >10 years experience)	Yes (conventional group: FODMAP reintroduction after 12-week elimination phase)	Participants asked to maintain routine activities; no other new treatments during study	IBS-SSS (primary, at 3 months; also at 1 and 6 months), IBS-QOL, WHO-5 well-being Index, CPSS, HADS - total, anxiety, depression subscales), adherence (patient-rated and dietitian-rated NRS), adverse events	IBS-SSS (modified with Likert scales for online use)	IBS-QOL; WHO-5 well-being index	Both Ayurvedic and conventional therapies (including low FODMAP) significantly reduced IBS-SSS at all time points vs. baseline (p < 0.001). Adjusted IBS-SSS reduction at 3 months was significantly greater for Ayurveda (123.8 pts) than conventional (72.7 pts); the difference was 51.1 pts (95% CI 3.8-98.5; p = 0.035), exceeding the MID of 50 pts. Both therapies contributed equally to outcome variance (treatment explained 68% overall). Patients' expectations (6.5%) and baseline severity (23.4%) also predicted outcome. QoL (IBS-QOL) improved in both groups with no significant between-group difference at 3 months. Secondary outcomes (anxiety, depression, WHO-5) improved significantly only in the Ayurveda group, except for IBS-QOL and WHO-5 in the conventional group. High adherence in both groups; no serious adverse events
	Krieger-Grübel et al., 2020 [33]	Randomized crossover RCT (2-arm)	3	21-day washout between crossover phases	Switzerland (Cantonal Hospital St. Gallen, outpatient)	Rome IV	Mixed (all subtypes with subjective lactose intolerance)	29	Median 30 (18-62) years	89.7% (26/29)	IBS-SSS mean: 209.97 ± 107.58 (6 pts severe >300)	LFD for 21 days: patients given a food-item table (high/low FODMAP content based on literature). Dietitian education at start; phone calls at days 21 and 65 to check adherence. Patients self-prepared meals	LLD for 21 days: patients received a table of product lactose content; instructed to eliminate lactose-containing foods and avoid hidden lactose sources. Same dietitian support	No	No pharmacologic agents to alter symptoms; no antibiotics within 4 weeks prior; no other specific diets	IBS-SSS (total and 5 subscores: pain severity, pain frequency, bloating severity, concern about bowel habits, impact on daily life), daily VAS (abdominal pain, bloating, well-being), stool frequency, stool consistency (Bristol Stool Chart), body weight, patient diet preference	IBS-SSS; daily VAS	None	Both LFD and LLD significantly reduced total IBS-SSS vs. baseline (LFD: −72 pts, p = 0.002; LLD: −57 pts, p = 0.007), with no significant difference between them (p = 0.67). Clinically relevant reduction ≥50 pts was achieved by 59.1% (LFD) and 54.2% (LLD). Daily VAS showed significant reductions in abdominal pain (p = 0.03) and bloating (p = 0.02) only with LFD, not LLD. Subscores for "concern about bowel habits" and "impact on daily life" improved with both diets. Stool frequency and consistency did not differ significantly. 12/25 patients preferred LLD for symptom control. Both diets considered equivalently effective on IBS-SSS; LFD had an edge on specific pain/bloating symptoms
	Liu et al., 2024 [34]	RCT (parallel, 4 arms)	4	4 (no post-diet follow-up)	China/tertiary care (West China Hospital, Sichuan University, Chengdu) - Western China	Rome IV	IBS-D, IBS-C, IBS-M	73 (PP; 80 randomized)	35.0 ± 9.8 (control); 34.9 ± 12.5 (LFD); 39.1 ± 9.5 (probiotics); 40.5 ± 11.0 (combined)	~52% (balanced across groups)	IBS-SSS: 202.8 ± 91.0 (control); 208.6 ± 57.3 (LFD); 254.6 ± 83.9 (probiotics); 243.9 ± 78.8 (combined)	LFD for 4 weeks based on a Chinese/Sichuan dietary background. Patients advised to limit cereals (wheat, rye), dairy, legumes, vegetables (garlic, onion, broccoli, etc.), and polyol sweeteners. Dietary photos submitted daily via mobile app for monitoring by dietitian	Three comparators: (1) control - conventional Chinese diet with TDA; (2) probiotics group - conventional diet + probiotic chewable tablets (Golden Bifidobacteria: *Bifidobacterium* ≥1×10⁷ CFU/g, *Lactobacillus bulgaricus* ≥1×10⁶ CFU/g, *Streptococcus thermophilus* ≥1×10⁶ CFU/g; 2g × 3/day); (3) combined - low FODMAP + probiotics	No	No other probiotics, prebiotics, or antibiotics permitted during study	IBS-SSS total and subscores (abdominal pain severity/frequency, bloating, bowel habits satisfaction, life impact); IBS-QOL; gut microbiota (16S rDNA V3-V4, Illumina MiSeq); fecal short-chain fatty acids (SCFAs); α- and β-diversity; LDA (LEfSe); Pearson correlation between microbiota and symptoms; daily dietary photos for compliance monitoring; FODMAP content of diet (g/day)	Symptom scale: IBS-SSS (0-500) + individual subscores (abdominal pain severity/frequency, bloating, bowel habit satisfaction, life impact)	QoL scale: IBS-QOL	This 4-arm RCT (n = 73 PP) in Western China confirmed the short-term efficacy of the LFD for IBS in the Sichuan (Chinese) dietary context. IBS-SSS significantly improved vs. baseline in all active groups at both 2 and 4 weeks: LFD (37.5% and 44.2% reductions), probiotics (51.4% and 62.0%), and combined (34.1% and 40.4%; all p < 0.05). At 4 weeks, both the LFD (116.0 ± 51.4) and probiotics group (125.5 ± 67.5) were significantly better than control (232.2 ± 113.7; p < 0.05), but the combined group did not reach statistical significance vs. control. Abdominal pain, pain frequency, bloating, bowel habit satisfaction, and life impact all improved significantly from baseline in all active groups. Gut microbiota analysis showed that LFD decreased Lachnoclostridium and Enterococcus, while the combined group increased Ruminococcus, Coprococcus, Akkermansia, and Ruminiclostridium. Pearson correlation identified Ruminiclostridium, Coprococcus, and Ruminococcus as negatively correlated with abdominal pain scores. No significant SCFA changes were detected
	Roth et al., 2024 [35]	Open-label RCT (non-inferiority)	4	24	Sweden (Skåne University Hospital, Malmö)	Rome IV	IBS-C, IBS-D, IBS-M, IBS-U, FBD	155	SSRD: 41 (29.5-53) years; FODMAP: 43 (33.8-56) years (median, IQR)	SSRD 80.5%; FODMAP 87.2%	Total IBS-SSS: SSRD 301 (233-348); FODMAP 300 (238-360) (median, IQR)	Low FODMAP: avoid fructans (wheat, onion, garlic), GOS (pulses), lactose (milk), excess fructose (honey), polyols (apples, pears, sugar-free gums). After 4 weeks: gradually reintroduce FODMAPs one by one	SSRD - avoid sucrose; restrict starch (bread, cereals, potato); increase meat, fish, eggs, dairy, certain fruits/vegetables. No reintroduction procedure needed	Yes (LFD)	Paracetamol, PPIs, allergy meds, hormonal contraceptives; vitamins/minerals in 35.5%; existing lactose-free/gluten-reduced diets	IBS-SSS (total and subscores), VAS-IBS (abdominal pain, diarrhea, constipation, bloating, nausea, daily-life impact, psychological well-being), extraintestinal Iextraintestinal BMI, waist circumference, blood pressure, sugar craving, saturation, responder rates	IBS-SSS; VAS-IBS	None	SSRD was non-inferior to low FODMAP at week 2 (79.2% vs 73.1%; p = 0.661) and week 4 (79.2% vs 78.2%; p = 1.000) for responder rates. Both diets significantly reduced all GI and extraintestinal variables from week 2 onward (p < 0.001 for most variables). SSRD produced greater reductions in weight (−1.6 vs −0.8 kg; p = 0.006), BMI (p = 0.005), and sugar craving (p = 0.05). ~25% of participants had no symptoms after 4 weeks. 40-50% did not meet IBS criteria at 6-month follow-up. Among participants who tested both diets, 64.7% preferred SSRD (easier to follow and better effect; p = 0.032)
	Russo et al., 2022 [36]	RCT (single-blind, parallel, 2 arms)	12	12 (no post-diet follow-up; multiple interim assessments at weeks 4 and 8)	Italy/outpatient gastroenterology (National Institute of Gastroenterology "S. de Bellis" Research Hospital, Castellana Grotte, Bari)	Rome III/Rome IV	IBS-D only	42 (21 LFD, 21 TBD; 72 randomized, 30 did not complete - PP analysis)	LFD: 64.7 ± 12.9 kg weight; TBD: 69.4 ± 12.9 kg (age NR explicitly; comparable groups)	LFD: 86% (18F/3M); TBD: 86% (18F/3M)	IBS-SSS: both groups moderate severity (IBS-SSS >125 required; baseline score NR explicitly, estimated 260-300 from graph); groups comparable at baseline	Personalized LFD for 12 weeks. Individual dietitian consultation at baseline; daily menus (3 meals + 2 snacks). Macronutrients: 50% carbohydrates, 30% lipids, 20% protein. Booklet with allowed/avoided/reduced foods (Monash University classification). 2-week run-in diet period. Food diaries kept; IDARS adherence scale completed at weeks 4, 8, 12	TBD for 12 weeks - all cereal products (bread, pasta, taralli salty biscuits, breakfast biscuits) prepared exclusively from Tritordeum flour (hybrid of durum wheat and wild barley). Same macronutrient targets and meal structure as LFD. IntiniFood provides Tritordeum products free monthly	No	Antipsychotic drugs and medications interfering with GI motility, weight, or appetite excluded; lactose-reduced diet allowed if constant throughout; alcohol and vigorous physical activity not permitted	IBS-SSS total score and 5 subscores (severity of abdominal pain, frequency of abdominal pain, severity of abdominal distension, dissatisfaction with bowel habits, interference with QoL) at weeks 0, 4, 8, 12; anthropometric measurements (weight, BMI, abdominal and waist circumferences) and BIA (BIA: fat mass, fat-free mass, total body water, extracellular water, phase angle, body cell mass); biochemical panel (glucose, lipid profile, CRP, vitamin D, iron, cobalamin, folate, creatinine, urea, urate, total protein); IDARS adherence scale; daily symptom and dietary diaries	Symptom scale: IBS-SSS (0-500) + 5 individual subscores at weeks 0, 4, 8, 12	QoL scale: not measured as a standalone QoL scale (anthropometric and biochemical outcomes were secondary; no IBS-QOL or SF-36 reported)	This single-blind Italian RCT (n = 42 completers; 21 LFD, 21 TBD) compared a 12-week LFD vs. a TBD in IBS-D patients. Both diets produced equivalent, clinically significant reductions in IBS-SSS total score across all time points (p < 0.0001 for both), with no significant between-group difference at any visit (diet × time interaction p>0.05 for all items). The initial drastic reduction occurred at week 4 (LFD: −95.0, 95% CI −37.7 to −152.3; TBD: −109.5, 95% CI −52.2 to −166.7), with improvement maintained through week 12 (LFD: −132.1, 95% CI −74.9 to −189.4; TBD: −130.5, 95% CI −73.2 to −187.7). All five IBS-SSS items (abdominal pain severity/frequency, distension, bowel habit dissatisfaction, QoL interference) significantly improved from baseline in both groups (p < 0.001 to p = 0.002), with no between-group differences. Anthropometric improvements (weight, BMI, waist/abdominal circumference, fat mass) were also equivalent between groups. Biochemically, fasting glucose and CRP decreased significantly over time (p = 0.003 and p = 0.02) with no group differences; vitamin D increased significantly (p = 0.04, time effect) with a trend favoring LFD but without significance. No changes in lipid profile, folate, cobalamin, or other micronutrients. The authors conclude that TBD represents a valid, equally effective alternative to LFD for IBS-D patients, with potential advantages in palatability and feasibility for Italian patients
	Singh et al., 2025 [37]	Randomized, double-blind, pilot feasibility trial (parallel, 2 arms)	4	4 (no post-treatment follow-up)	USA/two academic centers (University of Michigan; Beth Israel Deaconess Medical Center, Boston)	Rome IV	IBS-D only	26 randomized (11 LFD and 15 MD); 22 included in mITT; 20 completed and included in PP analysis (10 LFD and 10 MD)	NR (range 18-65 years)	NR	NR (abdominal pain NRS ≥3/10 required for inclusion)	LFD for 4 weeks - full FODMAP restriction (fructans, GOS, polyols, excess fructose, lactose all below Monash cutoffs). Meals provided by Epicured, New York. Research dietitian guidance	MD (FODMAP-simple diet for 4 weeks - restricted only fructans and galacto-oligosaccharides (below Monash cutoffs); polyols, fructose, and lactose NOT restricted (mean daily: polyols 0.86 g, lactose 1.37 g, excess fructose 0.99 g). Same epicured meal delivery. Double-blind design (same/similar dishes with FODMAPs added to FODMAP)-simple meals)	No	Stable doses of IBS medications permitted if no changes planned; no antibiotic or probiotic exclusion noted	Primary: FDA responder definition for abdominal pain intensity (≥30% reduction in weekly average of worst daily abdominal pain for ≥2/4 weeks); secondary: IBS-SSS (50-point and 100-point reduction); FDA responder for stool consistency (≥50% reduction in BSFS 6-7 days ≥2/4 weeks); FDA composite endpoint; responder for abdominal discomfort and bloating; IBS-QOL; 3-day food diaries (Nutritional Data System for Research); FODMAP intake; macronutrient/fiber intake	Symptom scale: IBS-SSS (0-500); FDA responder endpoint for abdominal pain intensity (NRS ≥30% reduction); FDA composite endpoint; BSFS stool consistency responder	QoL scale: IBS-QOL (reported as secondary outcome)	This double-blind, pilot feasibility RCT (n = 22 mITT; 20 PP) compared a traditional full LFD vs. a simplified FODMAP-simple diet (restricting only fructans and GOS) for 4 weeks in IBS-D patients. Using mITT analysis, 56.3% in the traditional LFD met the primary FDA endpoint for abdominal pain vs. 62.5% in the FODMAP-simple group (p = 0.71). Response rates for abdominal discomfort, bloating, and a 50-point reduction in IBS-SSS were also similar between groups (all p > 0.28). In the PP analysis, traditional LFD showed a significantly greater mean IBS-SSS reduction than FODMAP-simple (−174.6 ± 67.2 vs. −97.7 ± 73.5; p = 0.02), and a higher proportion achieved a 100-point IBS-SSS reduction (80% vs. 30.8%; p = 0.04). However, the stool consistency FDA endpoint showed a numerical trend favoring traditional LFD (50% vs. 14.3%; p = 0.09) due to polyol/fructose/lactose restriction. The FODMAP-simple diet had fewer dropouts due to side effects or compliance issues (12.5% vs. 26.3%), suggesting better tolerability. Authors conclude the simplified approach is feasible and well-tolerated, and that step-up restriction (adding FODMAP groups after initial response assessment) may optimize outcomes
	Tunali et al., 2024 [38]	Randomized, double-blind, parallel-group controlled trial	6	6	Turkey/multicenter (4 centers: Istanbul, Izmir, Kayseri)	Rome IV	IBS-C, IBS-D, IBS-M	121 (70 PD, 51 FODMAP)	PD: 35.9 ± 10.1; FODMAP: 37.9 ± 9.8	PD: 60%; FODMAP: 60.8%	IBS-SSS: PD 314.4 ± 92.8 (moderate-severe), FODMAP 276.8 ± 90.2	LFD: restricted fermentable carbs (fructans, GOS, lactose, excess fructose, polyols). Provided pamphlet (ACG guidelines) + 6-week menu plan by dietitian	Microbiome-based AI-assisted PD: foods scored 0-10 based on microbiome analysis to modulate gut microbiota. Dietitian-delivered with 6-week menu plan	No	No probiotics/prebiotics allowed; stable medications permitted	IBS-SSS total and subscores (pain severity/frequency, distension, bowel dissatisfaction, life interference); IBS-QOL; HADS (anxiety/depression); stool diary (Bristol); microbiome diversity (16S rRNA); dietary adherence	Symptom scale: IBS-SSS	QoL scale: IBS-QOL (reported as secondary outcome)	This multicenter RCT (n = 121; PD = 70, FODMAP = 51) compared a microbiome-based AI-assisted PD vs. the LFD over 6 weeks in IBS patients (Rome IV). Primary outcome (IBS-SSS change): PD −112.7 vs. LFD −99.9 (p = 0.29) - no significant difference between groups. Both groups showed significant within-group improvements in IBS-SSS (p < 0.001), symptom frequency, abdominal distension, and life interference (all p < 0.001). IBS-QOL improved significantly in both groups (p < 0.001). Anxiety (HADS) improved significantly in both groups (p < 0.001). Subgroup analysis: PD significantly reduced IBS-SSS in all subtypes (IBS-C p < 0.001, IBS-D p = 0.010, IBS-M p < 0.001); LFD improved IBS-SSS in IBS-C (p = 0.004) and IBS-M (p < 0.001) but not IBS-D (p = 0.312). PD improved IBS-QOL in all subtypes (IBS-C p < 0.001, IBS-D p < 0.001, IBS-M p = 0.008); LFD improved QOL only in IBS-C (p = 0.004) and IBS-D (p = 0.022); no improvement in IBS-M (p = 0.646). Microbiome: PD led to significant increases in alpha diversity (Shannon) and beta diversity shifts (p < 0.05); LFD showed no significant change in alpha diversity. Faecalibacterium prausnitzii increased significantly in the PD group (p < 10⁻⁴) but not in the LFD group. Ruminococcaceae decreased in PD (p < 10⁻⁵) but not in LFD. Conclusion: PD demonstrates comparable overall symptom relief to LFD but with broader subtype coverage, greater QOL benefit in IBS-M, and more favorable microbiome effects

A total of 23 RCTs [8,12,18-38] were included in this meta-analysis, encompassing 2,164 randomized participants and 1,852 completed participants across all intervention arms. Sample sizes ranged from 29 to 155 participants per study. Across the included trials, participants were allocated to a range of comparator conditions, including traditional dietary advice, habitual diet, higher FODMAP diet, and other active dietary interventions not aligned with NICE, reflecting the variability of control conditions employed in the dietary management of IBS.

IBS Symptom Severity Score

Low FODMAP vs. traditional dietary advice: Seven studies (n = 324 participants) [8,18,20-24] compared the low FODMAP diet to traditional dietary advice in terms of IBS-SSS. The pooled analysis demonstrated a statistically significant reduction in symptom severity favoring the low FODMAP diet (SMD: −0.71; 95% CI (−1.02, −0.40), p < 0.00001). Pooled studies showed substantial heterogeneity (I² = 67%) (Figure 2).

**Figure 2:**
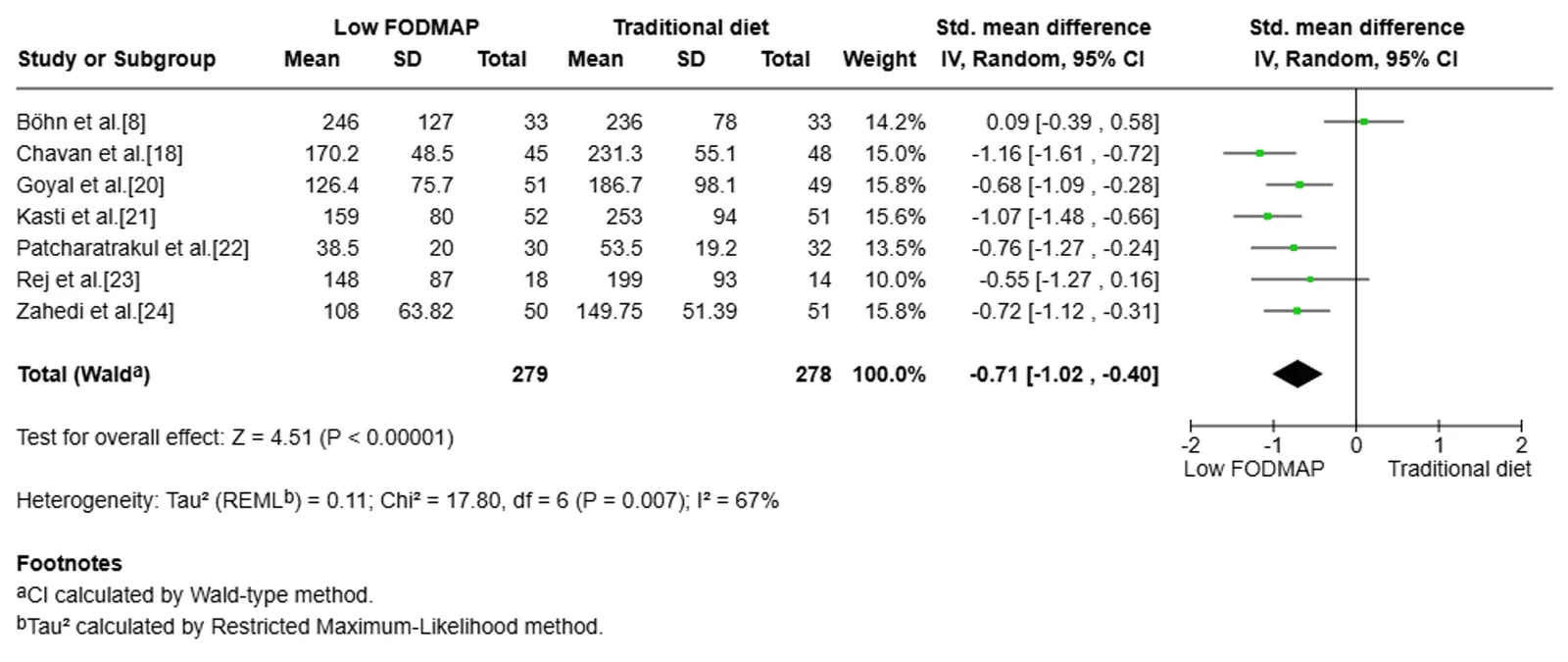
Forest plot of low FODMAP vs. traditional diet CI: confidence interval; df: degrees of freedom; FODMAP: fermentable oligosaccharides, disaccharides, monosaccharides, and polyols; IV: inverse variance; REML: restricted maximum likelihood; SD: standard deviation; Std.: standardized

In a post-hoc sensitivity analysis excluding Bohn et al. [8], the pooled SMD was -0.85 (95% CI -1.04 to -0.66; p < 0.001), with heterogeneity reduced from I² = 67% to I² = 0%.

Low FODMAP vs. habitual diet: Three studies (n = 202 participants) [26-28] compared the low FODMAP diet to a habitual diet in terms of IBS-SSS. The pooled analysis demonstrated a statistically significant reduction in symptom severity favoring the low FODMAP diet (SMD: −0.53; 95% CI (−0.82, −0.25), p = 0.0002). Heterogeneity was low (I² = 27%) (Figure 3).

**Figure 3:**
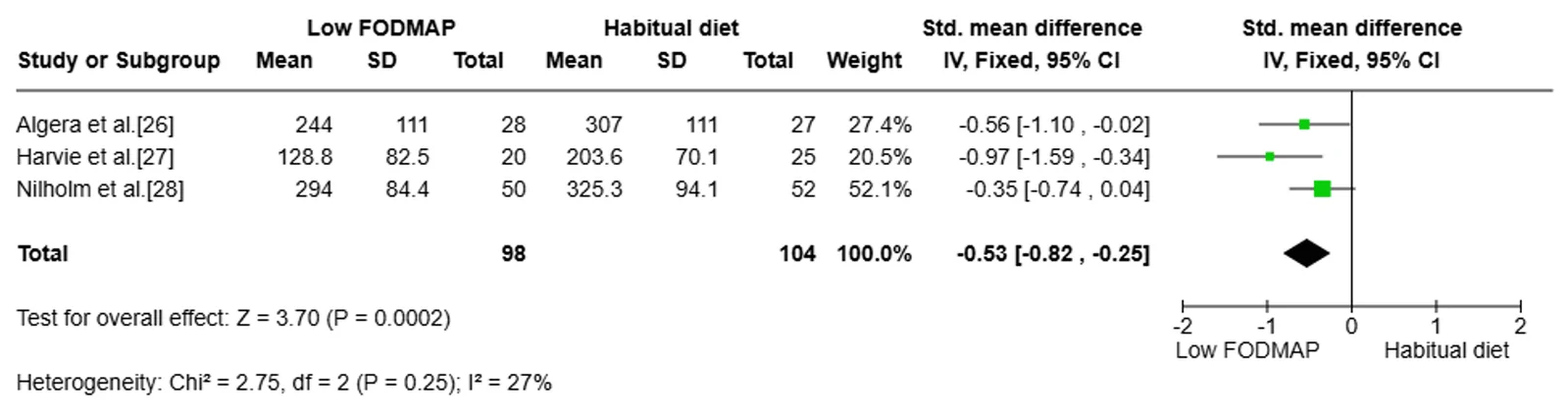
Forest plot of low FODMAP vs. habitual diet CI: confidence interval, FODMAP: fermentable oligosaccharides, disaccharides, monosaccharides, and polyols, IV: inverse variance, SD: standard deviation, Std.: standardized

Low FODMAP vs. higher FODMAP diet: Two studies (n = 47 participants) [29,30] compared the low FODMAP diet to a higher FODMAP diet. The pooled analysis demonstrated a statistically significant reduction in IBS-SSS favoring the low FODMAP diet (SMD: −0.87; 95% CI (−1.47, −0.26), p = 0.005). No statistical heterogeneity was detected (I² = 0%), although the small number of contributing studies (two trials) should be considered when interpreting this result (Figure 4).

**Figure 4:**
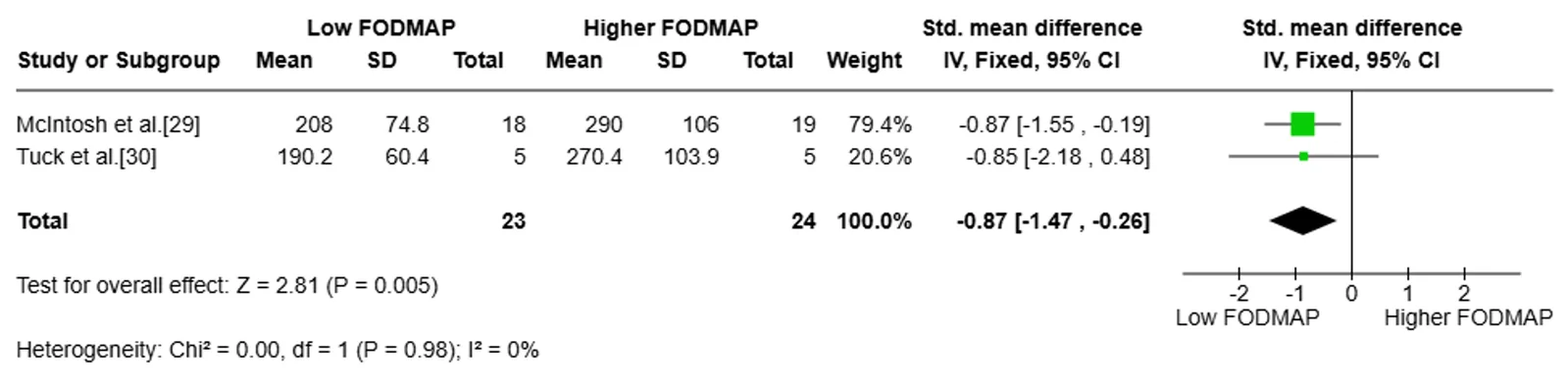
Forest plot of low FODMAP vs. higher FODMAP diet CI: confidence interval, FODMAP: fermentable oligosaccharides, disaccharides, monosaccharides, and polyols, IV: inverse variance, SD: standard deviation, Std.: standardized

Low FODMAP vs. other active dietary interventions (non-NICE): Seven studies (n = 486 participants) [31-35,37,38] compared the low FODMAP diet to other active dietary interventions not aligned with NICE. The pooled analysis showed no statistically significant difference in IBS-SSS between groups (SMD: −0.64; 95% CI (−1.58, 0.29), p = 0.18). Pooled studies showed considerable heterogeneity (I² = 95%).

In a post-hoc sensitivity analysis excluding Jeitler et al. [32], whose effect estimate was a marked outlier, and Singh et al. [37], the only study in this subgroup to report a statistically significant effect favoring the comparator intervention over the low FODMAP diet, the pooled analysis yielded an SMD of -0.39 (95% CI -0.71 to -0.07; p = 0.0180), with I² = 53.3% (Figure 5).

**Figure 5:**
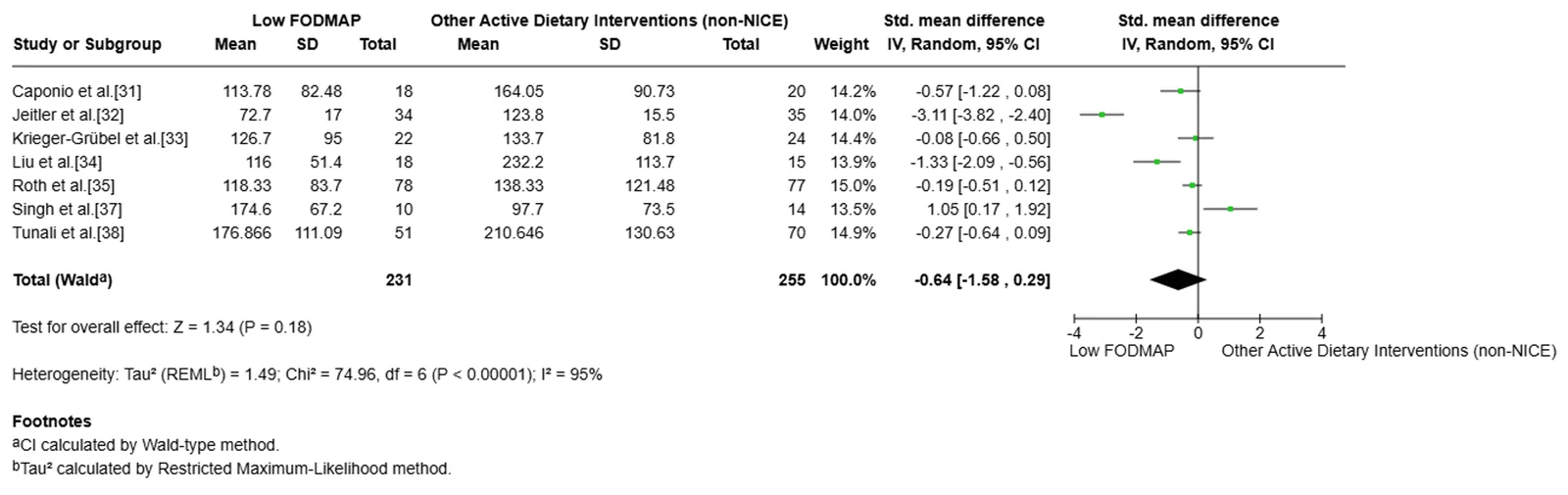
Forest plot of low FODMAP vs. other active dietary interventions CI: confidence interval, FODMAP: fermentable oligosaccharides, disaccharides, monosaccharides, and polyols, IV: inverse variance, NICE: National Institute for Health and Care Excellence, REML: restricted maximum likelihood, SD: standard deviation, Std.: standardized

Adequate Relief/Global Symptom Response

Low FODMAP vs. traditional dietary advice: Seven studies (n = 612 participants) [18-23,25] compared the low FODMAP diet with traditional dietary advice for adequate relief or global symptom response. The pooled analysis demonstrated a statistically significant benefit favoring the low FODMAP diet (OR: 2.15; 95% CI (1.54, 3.01), p < 0.00001). No heterogeneity was detected between the pooled studies (I² = 0%) (Figure 6).

**Figure 6:**
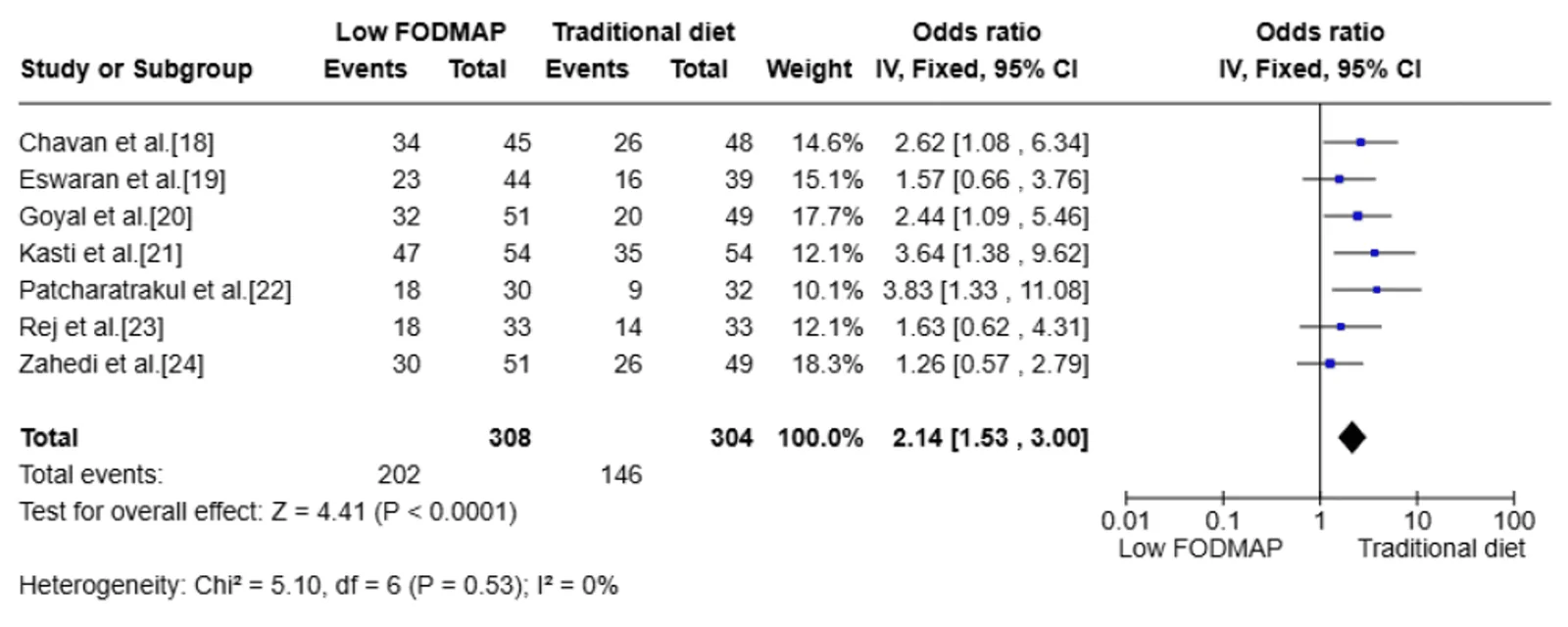
Forest plot of responder rates (low FODMAP vs. traditional diet) CI: confidence interval, FODMAP: fermentable oligosaccharides, disaccharides, monosaccharides, and polyols, IV: inverse variance

Low FODMAP vs. habitual diet: Three studies (n = 250 participants) [12,26,28] compared the low FODMAP diet to a habitual diet for adequate relief or global symptom response. The pooled analysis demonstrated a statistically significant benefit favoring the low FODMAP diet (OR: 4.75; 95% CI (2.41, 9.34), p < 0.00001). Moderate heterogeneity was observed between the pooled studies (I² = 45%) (Figure 7).

**Figure 7:**
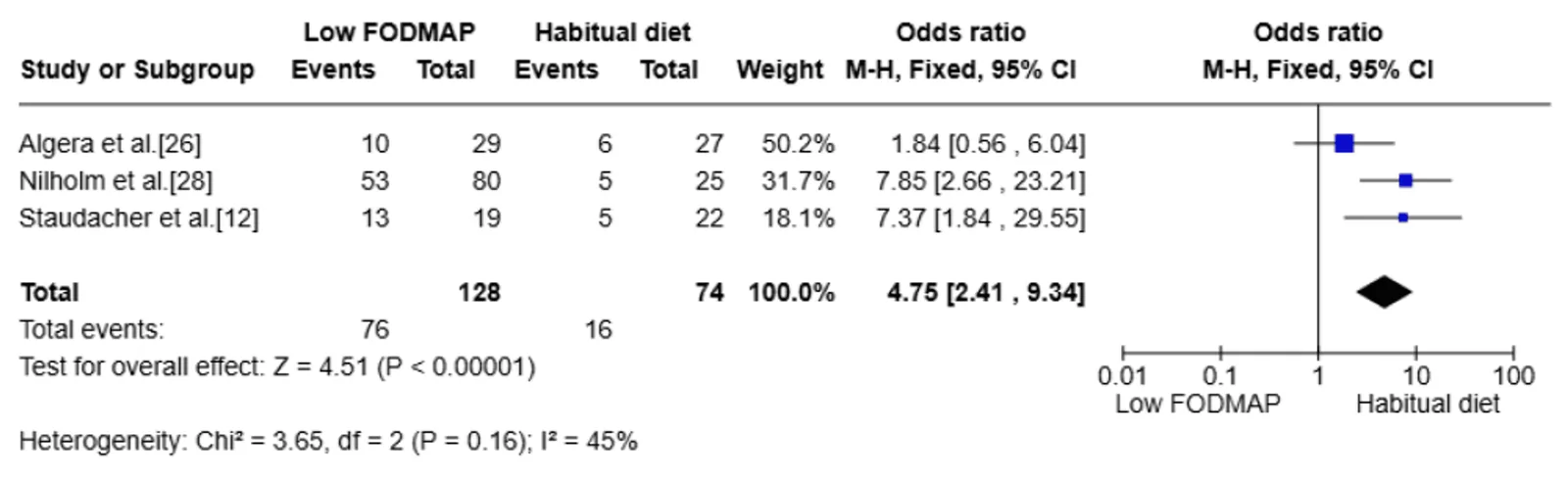
Forest plot of responder rates (low FODMAP vs. habitual diet) CI: confidence interval, FODMAP: fermentable oligosaccharides, disaccharides, monosaccharides, and polyols, M-H: Mantel-Haenszel

Low FODMAP vs. other active dietary interventions (non-NICE): Three studies (n = 221 participants) [33,35,37] compared the low FODMAP diet to other active dietary interventions. The pooled analysis did not demonstrate a statistically significant difference in adequate relief or global symptom response between groups (OR: 0.83; 95% CI (0.46, 1.50), p = 0.54). No heterogeneity was detected between the pooled studies (I² = 0%) (Figure 8).

**Figure 8:**
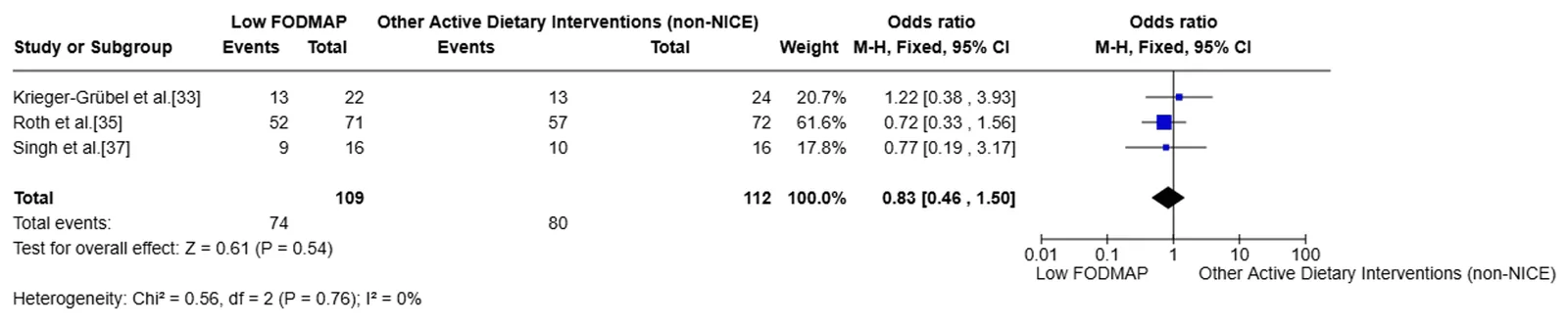
Forest plot of responder rates (low FODMAP vs. other active dietary interventions) CI: confidence interval, FODMAP: fermentable oligosaccharides, disaccharides, monosaccharides, and polyols, M-H: Mantel-Haenszel, NICE: National Institute for Health and Care Excellence

No eligible studies comparing the low FODMAP diet with a higher FODMAP diet reported adequate relief or global symptom response in a format suitable for meta-analysis.

Abdominal Pain Severity Score

Low FODMAP vs. traditional dietary advice: Six studies (n = 444 participants) [8,19,20,22-24] compared the low FODMAP diet to traditional dietary advice on abdominal pain severity. The pooled analysis demonstrated a statistically significant reduction in abdominal pain favoring the low FODMAP diet (SMD: −0.35; 95% CI (−0.54, −0.16), p = 0.0003). Heterogeneity was minimal (I² = 10%) (Figure 9).

**Figure 9:**
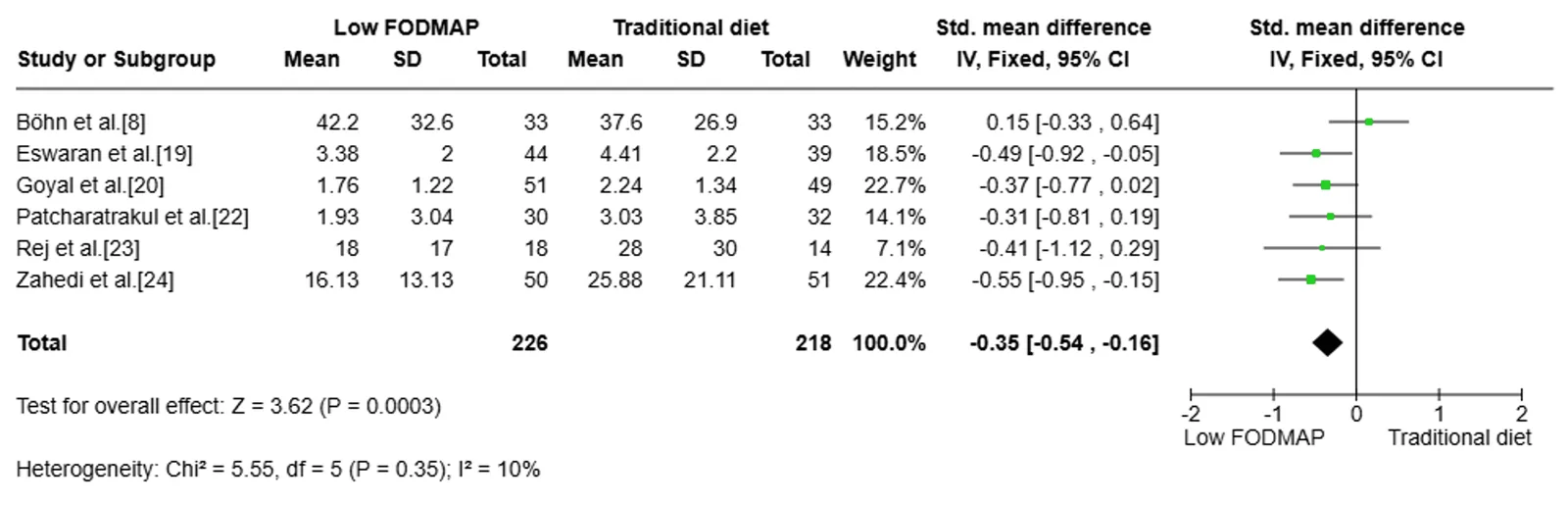
Forest plot of symptom severity scores (low FODMAP vs. traditional diet) CI: confidence interval, FODMAP: fermentable oligosaccharides, disaccharides, monosaccharides, and polyols, IV: inverse variance, SD: standard deviation, Std.: standardized

Low FODMAP vs. habitual diet: Two studies (n = 157 participants) [26,28] compared the low FODMAP diet with a habitual diet for abdominal pain severity. The pooled estimate favored the low FODMAP diet, but the result was at the threshold of statistical significance (SMD: −0.32; 95% CI (−0.63, −0.00), p = 0.05). No heterogeneity was detected between the pooled studies (I² = 0%) (Figure 10).

**Figure 10:**
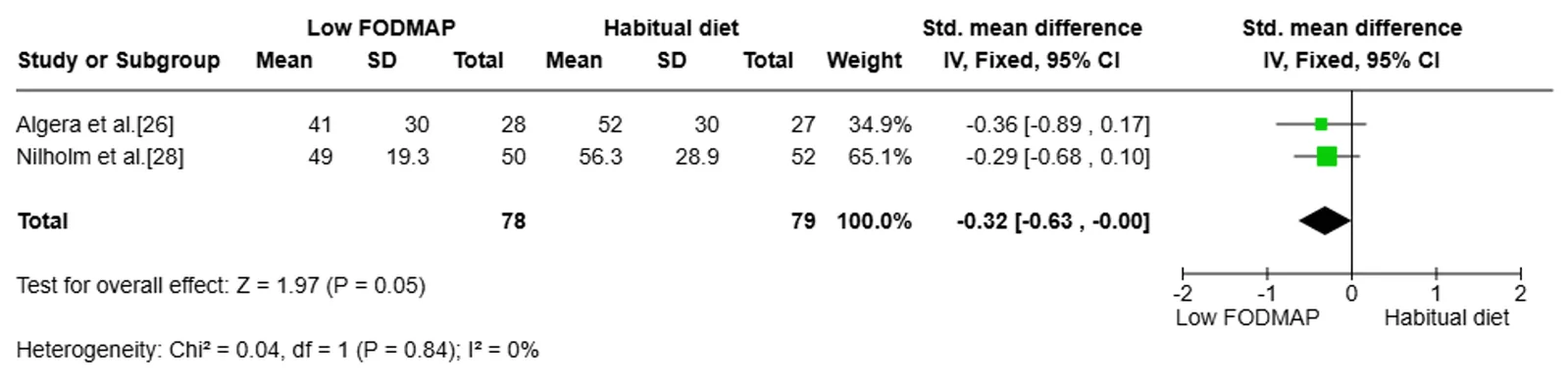
Forest plot of symptom severity scores (low FODMAP vs. habitual diet) CI: confidence interval, FODMAP: fermentable oligosaccharides, disaccharides, monosaccharides, and polyols, IV: inverse variance, SD: standard deviation, Std.: standardized

Low FODMAP vs. other active dietary interventions (non-NICE): Five studies (n = 397 participants) [33-36,38] compared the low FODMAP diet to other active dietary interventions in terms of abdominal pain severity. The pooled analysis showed no statistically significant difference between groups (SMD: −0.18; 95% CI (−0.38, 0.02), p = 0.08). The point estimate favored the low FODMAP diet, although the difference was not statistically significant. Heterogeneity was low across the pooled studies (I² = 17%) (Figure 11).

**Figure 11:**
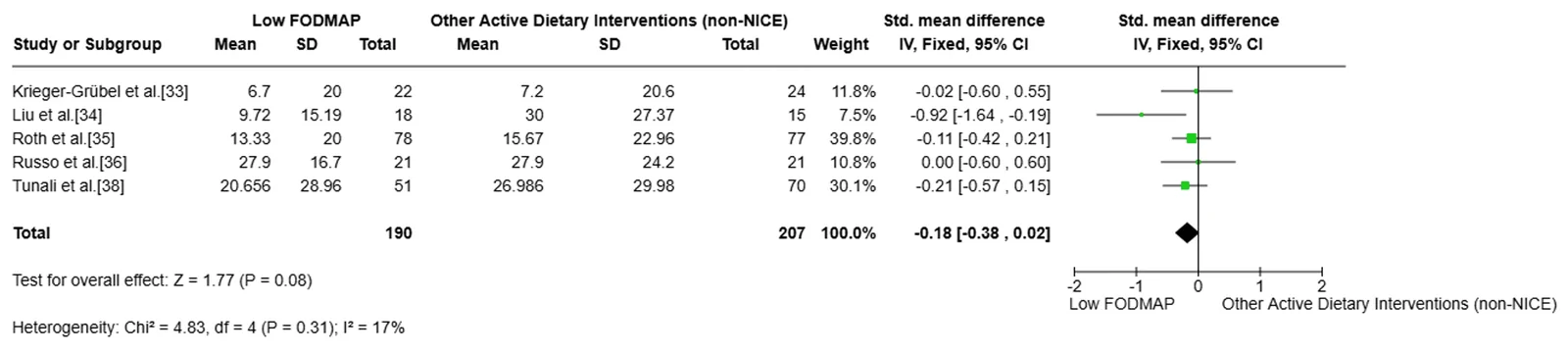
Forest plot of symptom severity scores (low FODMAP vs. other active dietary interventions) CI: confidence interval, FODMAP: fermentable oligosaccharides, disaccharides, monosaccharides, and polyols, IV: inverse variance, NICE: National Institute for Health and Care Excellence, SD: standard deviation, Std.: standardized

No eligible studies comparing the low FODMAP diet with a higher FODMAP diet reported abdominal pain severity in a format suitable for meta-analysis.

Bloating/Distension Score

Low FODMAP vs. traditional dietary advice: Six studies (n = 444 participants) [8,19,20,22-24] compared the low FODMAP diet to traditional dietary advice on bloating or distension severity. The pooled analysis demonstrated a statistically significant reduction in bloating favoring the low FODMAP diet (SMD: −0.45; 95% CI (−0.75, −0.15), p = 0.003). Moderate to substantial heterogeneity was observed between the pooled studies (I² = 59%) (Figure 12).

**Figure 12:**
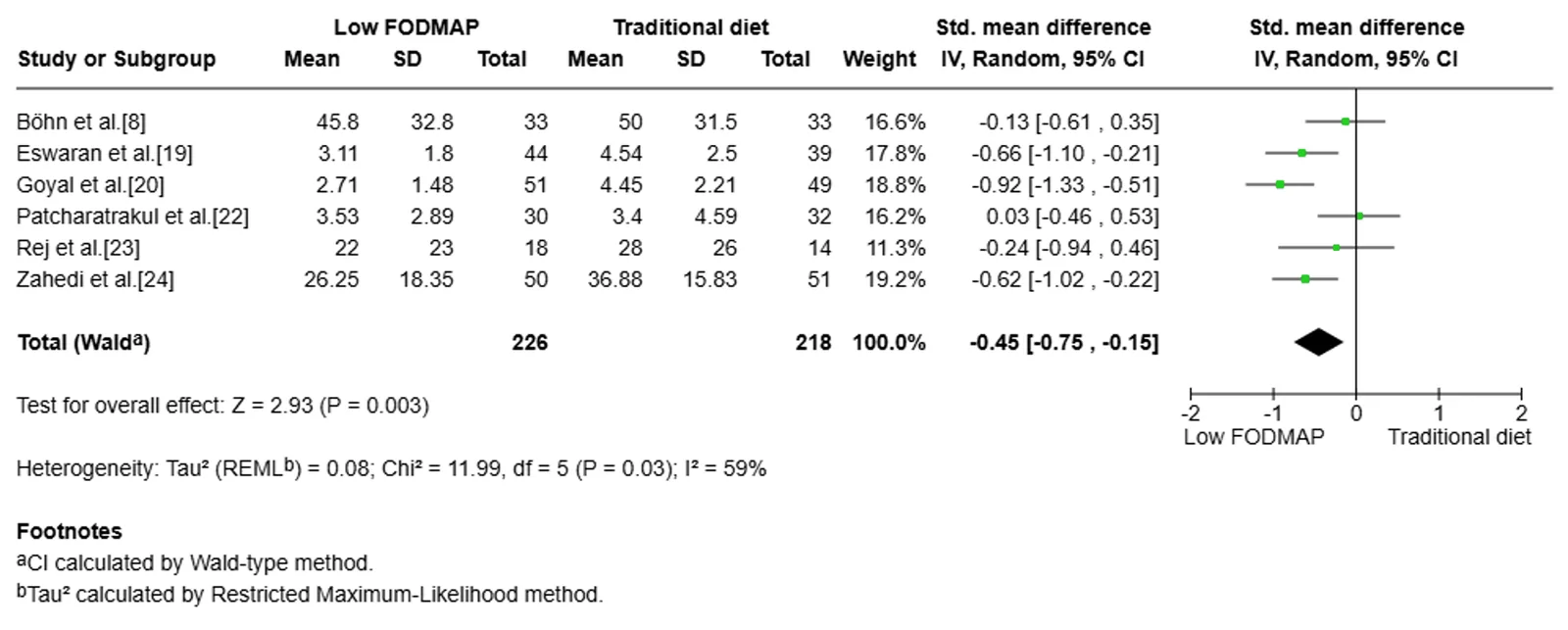
Forest plot of bloating severity scores (low FODMAP vs. traditional diet) CI: confidence interval, FODMAP: fermentable oligosaccharides, disaccharides, monosaccharides, and polyols, IV: inverse variance, REML: restricted maximum likelihood, SD: standard deviation, Std.: standardized

In a post-hoc sensitivity analysis excluding Goyal et al. [20], the pooled SMD was -0.35 (95% CI -0.64 to -0.06; p = 0.0168), with heterogeneity reduced from I² = 59% to I² = 40%.

Low FODMAP vs. habitual diet: Two studies (n = 157 participants) [26,28] compared the low FODMAP diet with a habitual diet for bloating or distension severity. The pooled analysis demonstrated a statistically significant reduction in bloating favoring the low FODMAP diet (SMD: −0.49; 95% CI (−0.81, −0.17), p = 0.002). No heterogeneity was detected between the pooled studies (I² = 0%) (Figure 13).

**Figure 13:**
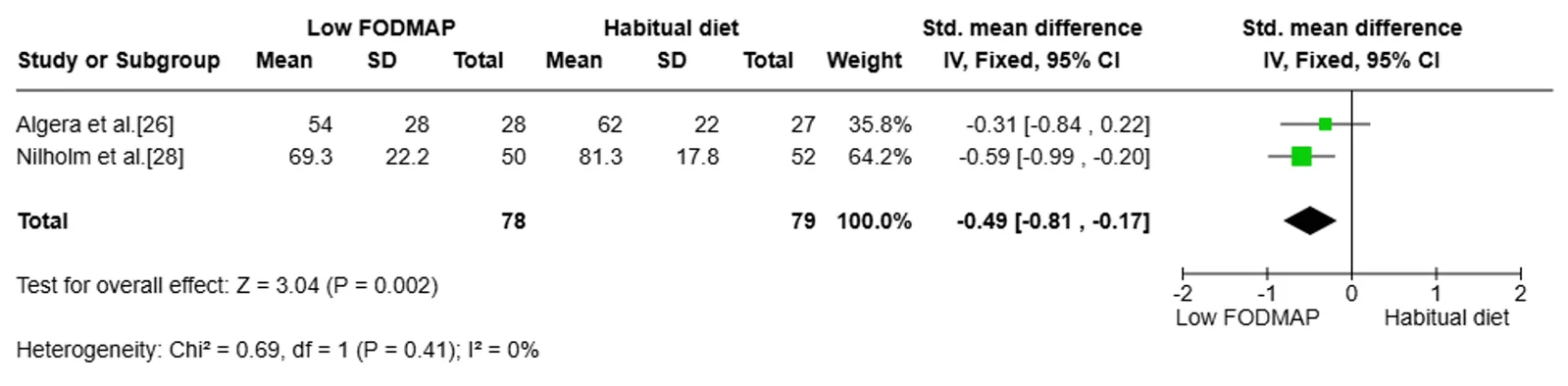
Forest plot of bloating severity scores (low FODMAP vs. habitual diet) CI: confidence interval, FODMAP: fermentable oligosaccharides, disaccharides, monosaccharides, and polyols, IV: inverse variance, SD: standard deviation, Std.: standardized

Low FODMAP vs. other active dietary interventions (non-NICE): Five studies (n = 397 participants) [33-36,38] compared the low FODMAP diet to other active dietary interventions in terms of bloating or distension severity. The pooled analysis demonstrated a statistically significant reduction in bloating favoring the low FODMAP diet (SMD: −0.22; 95% CI (−0.42, −0.02), p = 0.03). Pooled studies showed low-to-moderate heterogeneity (I² = 34%) (Figure 14).

**Figure 14:**
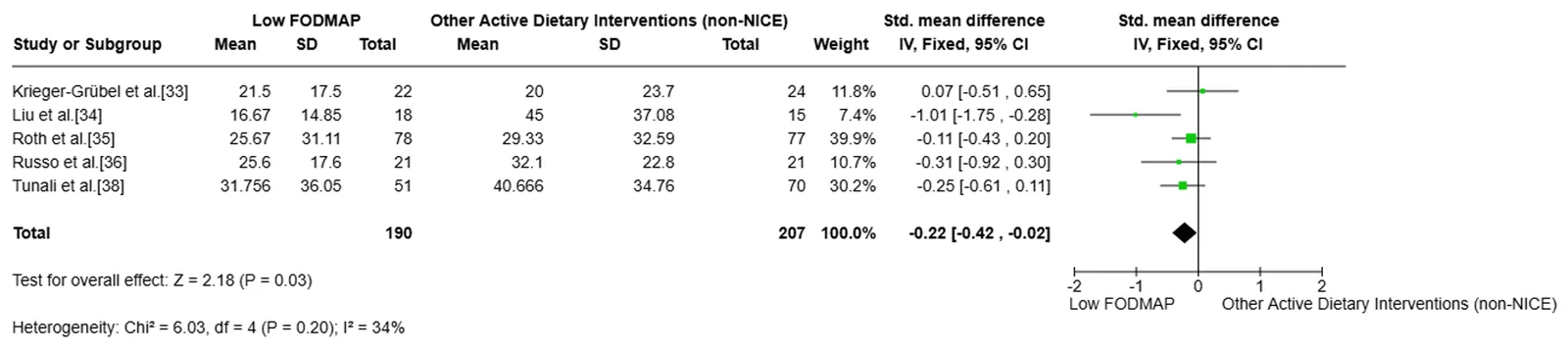
Forest plot of bloating severity scores (low FODMAP vs. other active dietary interventions) CI: confidence interval, df: degrees of freedom, FODMAP: fermentable oligosaccharides, disaccharides, monosaccharides, and polyols, IV: inverse variance, NICE: National Institute for Health and Care Excellence, SD: standard deviation, Std.: standardized

No eligible studies comparing the low FODMAP diet with a higher FODMAP diet reported bloating or distension severity in a format suitable for meta-analysis.

QoL Score

Low FODMAP vs. traditional dietary advice: Four studies (n = 362 participants) [18,20,21,23] compared the low FODMAP diet to traditional dietary advice in terms of QoL. The pooled analysis demonstrated a statistically significant improvement in QoL favoring the low FODMAP diet (SMD: 0.48; 95% CI (0.27, 0.69), p < 0.00001). There was low-to-moderate heterogeneity between the pooled studies (I² = 42%) (Figure 15).

**Figure 15:**
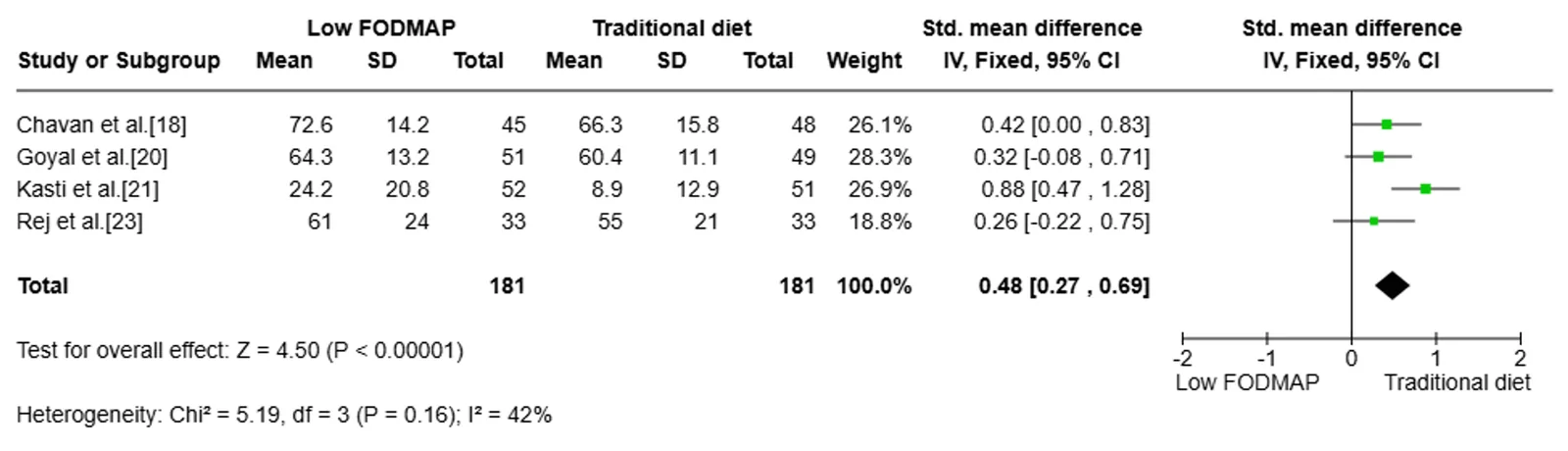
Forest plot of quality-of-life scores (low FODMAP vs. traditional dietary advice) CI: confidence interval, FODMAP: fermentable oligosaccharides, disaccharides, monosaccharides, and polyols, IV: inverse variance, SD: standard deviation, Std.: standardized

Low FODMAP vs. other active dietary interventions (non-NICE): Three studies (n = 223 participants) [32,36,38] compared the low FODMAP diet to other active dietary interventions not aligned with NICE guidelines for QoL. The pooled analysis showed no statistically significant difference in QoL between groups (SMD: −0.13; 95% CI (−0.40, 0.13), p = 0.33). No heterogeneity was detected between the pooled studies (I² = 0%) (Figure 16).

**Figure 16:**
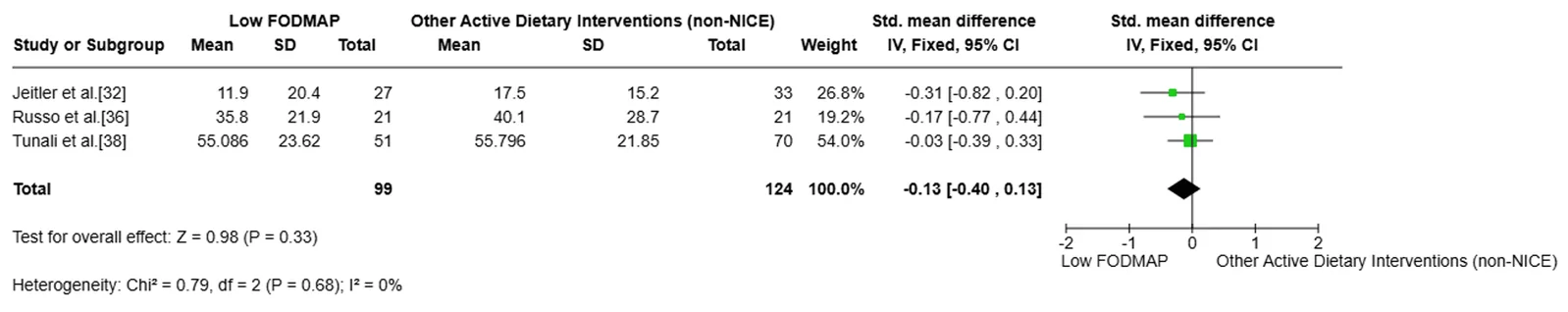
Forest plot of quality-of-life scores (low FODMAP vs. other active dietary interventions) CI: confidence interval, FODMAP: fermentable oligosaccharides, disaccharides, monosaccharides, and polyols, IV: inverse variance, NICE: National Institute for Health and Care Excellence, SD: standard deviation, Std.: standardized

No eligible studies comparing the low FODMAP diet with a higher FODMAP diet reported quality-of-life outcomes in a format suitable for meta-analysis.

Risk of Bias Assessment

Risk of bias was assessed across 23 included studies [8,12,18-38] using the Cochrane RoB 2 tool. For the randomization process (D1), 22 studies [8,12,18-29,31-38] were rated low risk, one [30] raised some concerns, and none were high risk. For allocation concealment (D2), 15 studies [8,20,21,23,25-29,32-35,37,38] were rated low risk, seven had some concerns [12,18,22,24,30,31,36], and one [19] was high risk. The most consistently problematic domain was blinding of participants and personnel (D3), largely because dietary trials make blinding participants and personnel inherently difficult. Only three studies [26,37,38] achieved a low-risk rating here, while one study [29] had some concerns, and the remaining 19 studies [8,12,18-25,27,28,30-36] were deemed high risk. Blinding of outcome assessment (D4) was rated low risk in five studies [19,21,25,26,37], some concerns in 16 [8,12,18,20,22-24,28-31,33-36,38], and high risk in two [27,32]. For incomplete outcome data (D5), 13 studies were low risk [8,18-21,23-26,29,34,35,38], seven had some concerns [12,22,27,28,31-33], and three [30,36,37] were high risk. Selective reporting (D6) was low risk across 22 studies [8,12,18-29,31-38], while Tuck et al. [30] was high risk. Applying the RoB algorithm, only Algera et al. [26] and Tunali et al. [38] were judged as having an overall low risk of bias, while Eswaran et al. [19], Harvie et al. [27], Jeitler et al. [32], Russo et al. [36], and Tuck et al. [30] were rated overall high risk. The remaining 16 studies [8,12,18,20-25,28,29,31,33-35,37] were rated overall as some concerns, driven predominantly by the blinding of participants and personnel (D3) domain. This high proportion of studies with unclear or high risk of bias warrants caution when interpreting the pooled estimates, especially given that several of these studies contribute substantial weight to individual subgroup analyses (Figures 17-18).

**Figure 17:**
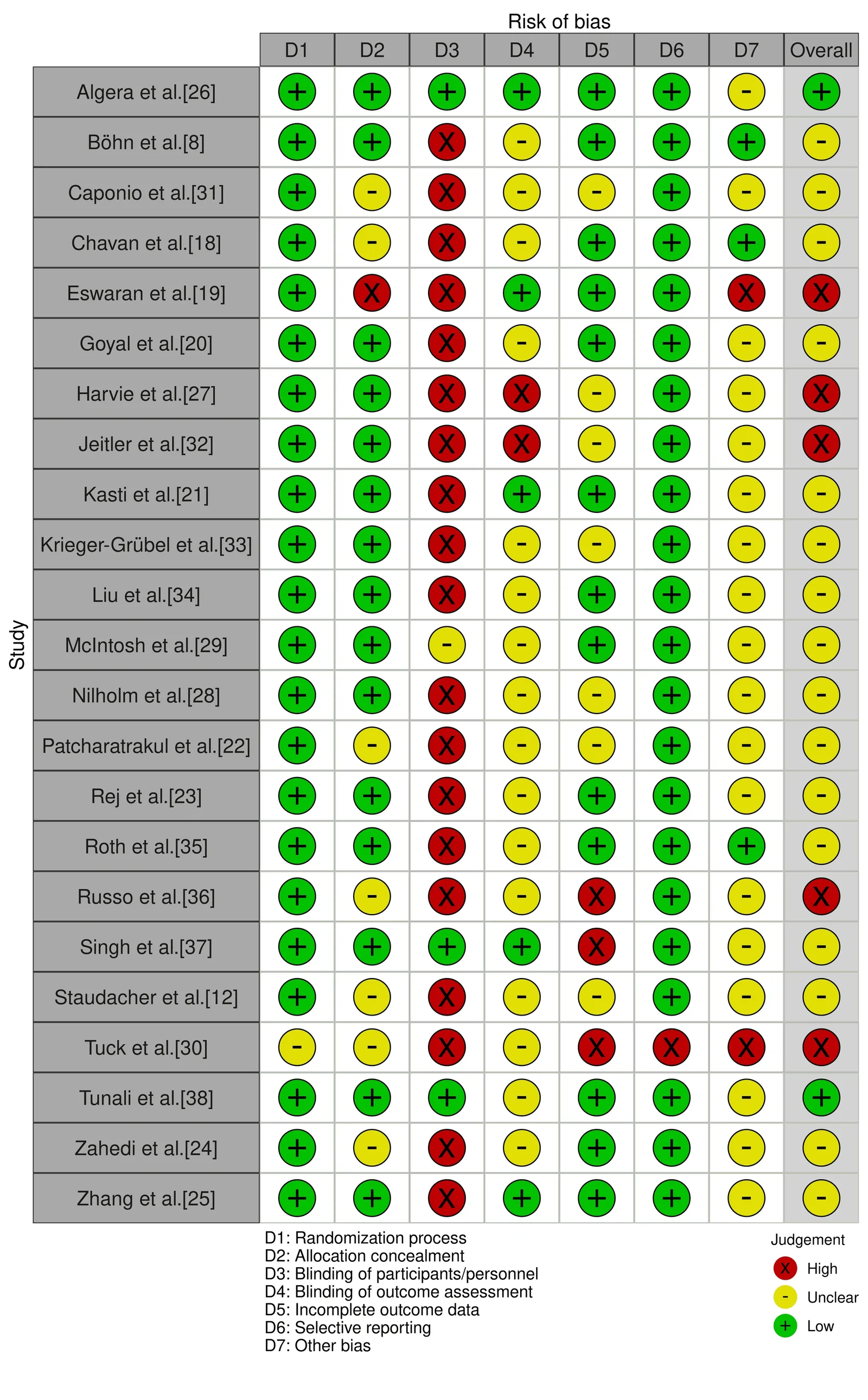
Risk of bias summary

**Figure 18:**
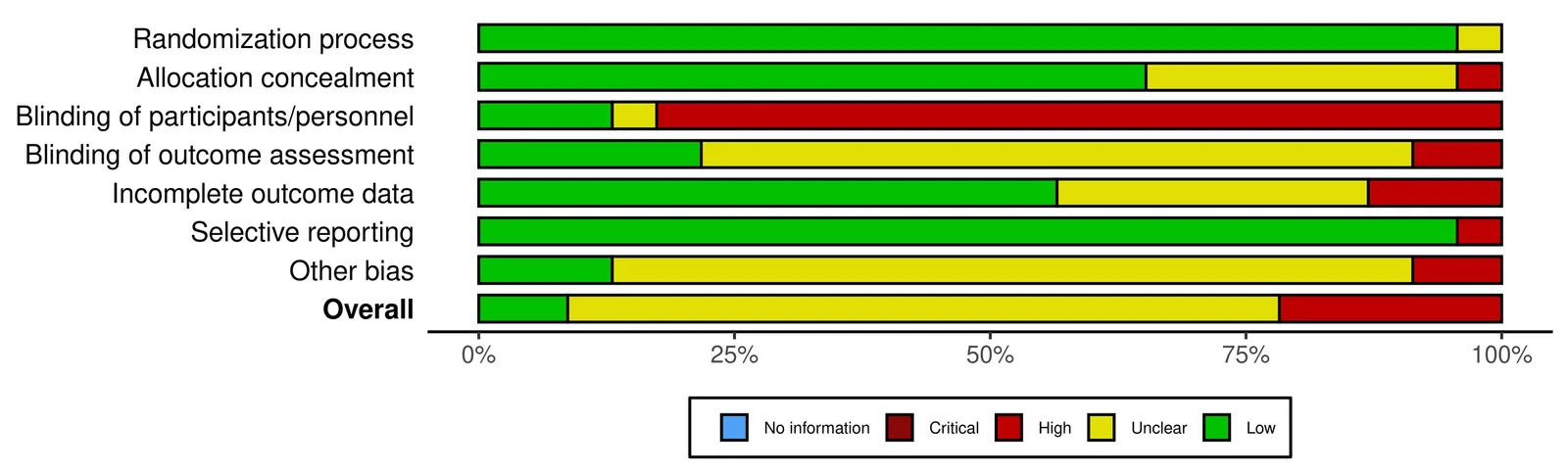
Risk of bias graph

Publication Bias

Publication bias could not be formally assessed because no pooled comparison included 10 or more studies (maximum: seven), the minimum recommended in the Cochrane Handbook for reliable funnel plot asymmetry detection and for Egger's regression to have adequate statistical power. Visual inspection of funnel plots was therefore considered insufficient for meaningful interpretation, as apparent asymmetry with few studies may reflect between-study heterogeneity, small-study effects, or chance rather than true publication bias. Accordingly, funnel plots are not presented, and the possibility of publication bias or small-study effects cannot be excluded.

Discussion

Principal Findings

This meta-analysis of 23 RCTs encompassing 2,164 randomized participants (1852 completers) provides a comprehensive evaluation of the low FODMAP diet compared with multiple control conditions in patients with IBS. Consistent with the pre-specified outcome hierarchy, IBS-SSS is discussed below as the primary efficacy outcome, with adequate relief, abdominal pain, bloating, and QoL considered as secondary outcomes. Collectively, the findings show that the low FODMAP diet confers clinically meaningful and statistically significant benefits over passive or less structured comparators, including traditional dietary advice and habitual diet, across a range of patient-relevant outcomes. However, compared with other structured active dietary interventions, the low FODMAP diet does not emerge as clearly superior, a pattern consistent with the emerging literature.

Comparison With Previous Evidence

The present analysis demonstrated a significant reduction in IBS-SSS favoring the low FODMAP diet over traditional dietary advice (SMD: −0.71) and habitual diet (SMD: −0.53), with the most pronounced effect observed against a higher FODMAP diet (SMD: −0.87). These findings closely align with the umbrella review by Bogdanowska-Charkiewicz et al., which synthesized 16 meta-analyses including 9,904 patients and reported a pooled SMD of −0.599 for IBS-SSS reduction, reinforcing the robustness of our estimates across a larger and more diverse evidence base [40]. Similarly, van Lanen et al. reported a moderate-to-large reduction in IBS severity versus control diets (SMD: −0.66), consistent with the effect size observed in the present analysis [41].

For global symptom response or adequate relief, the low FODMAP diet showed more than a twofold advantage over traditional dietary advice (OR: 2.15) and nearly a fivefold advantage over habitual diet (OR: 4.75). These estimates align with Wang et al., who reported significantly improved global IBS symptoms with the low FODMAP diet compared with normal or usual dietary controls [42]. The markedly higher ORs against habitual diet, compared with traditional advice, may reflect the active nature of the NICE-aligned dietary counseling provided in the traditional advice arm, suggesting that even structured first-line dietetic advice captures some, but not all, of the benefit attributable to FODMAP restriction.

Interpretation and Possible Explanations

The absence of a statistically significant difference in IBS-SSS and adequate relief when the low FODMAP diet was compared with other active dietary interventions (non-NICE) warrants careful consideration. The considerable heterogeneity (I² = 95%) in the IBS-SSS subgroup, largely attributable to outlier studies including Singh et al. [37], which uniquely favored the comparator, and Jeitler et al. [32], complicates direct interpretation. A 2024 network meta-analysis published in The Lancet Gastroenterology and Hepatology similarly concluded, with high confidence, that no dietary intervention demonstrated superiority over others. However, the low FODMAP diet had the most evidence supporting its use [43]. This emerging body of evidence suggests that the low FODMAP diet is not uniquely effective but rather the best-evidenced representative of a broader class of structured dietary therapies.

The low FODMAP diet produced statistically significant reductions in abdominal pain versus traditional advice (SMD: −0.35) and a near-significant reduction versus habitual diet (SMD: −0.32). However, it showed no significant advantage over active dietary comparators (SMD: −0.18, p = 0.08). These effect sizes warrant interpretation beyond statistical significance alone: an SMD in the region of −0.3 to −0.35 corresponds to a small-to-moderate effect by conventional benchmarks, and whether it meets a clinically important difference for IBS-related abdominal pain depends on the specific instrument's established minimal clinically important difference, which was not uniformly reported across the included trials. Statistical significance in a well-powered pooled analysis does not by itself establish that patients would perceive the reduction as meaningful, and this distinction should temper the clinical weight placed on these findings. These findings are notable when considered alongside the umbrella review by Bogdanowska-Charkiewicz et al., which reported no significant pooled effect of the LFD on abdominal pain across meta-analyses [40]. This discrepancy may reflect our more granular stratification by comparator type, which shows that the low FODMAP diet advantage for pain is apparent primarily against less structured control conditions rather than structured dietary comparators. The non-significant result against active comparators (p = 0.08) may represent a genuine but modest effect that is underpowered in the current subgroup; it should not be interpreted as evidence of equivalence and warrants further adequately powered investigation.

For bloating and distension, the low FODMAP diet showed significant reductions across all three comparator subgroups, including other active dietary interventions (SMD: −0.22, p = 0.03). This is a clinically important finding, as bloating ranks among the most debilitating and treatment-resistant IBS symptoms [44]. However, an SMD of −0.22 versus active comparators is small, and its clinical relevance to individual patients should be weighed separately from its statistical significance, particularly given that this subgroup includes some of the more heterogeneous comparator conditions in the review. The lowFODMAP diet retained a statistically significant advantage over active comparators for bloating, even when it did not for global or pain outcomes, which is plausible given the direct mechanistic link between fermentable carbohydrate fermentation, gas production, and bloating symptoms [43].

QoL improved significantly with the low FODMAP diet versus traditional dietary advice (SMD: 0.48), corroborating findings from the umbrella review, which reported a pooled SMD of 0.259 across five meta-analyses [40]. However, it did not demonstrate a QoL benefit over other active interventions (SMD: −0.13, p = 0.33). The lack of a significant QoL difference between the low FODMAP diet and other structured dietary interventions suggests that factors common to structured dietary interventions, such as dietary education, patient engagement, and therapeutic support, may contribute to observed improvements. However, the present analysis cannot separate these factors from diet-specific effects. Potential mechanisms such as increased dietary agency, therapeutic alliance, and symptom attribution are plausible contributors, though the present data cannot establish their relative contribution, nor can they be interpreted as showing that FODMAP restriction itself is clinically inert; both structural and diet-specific mechanisms may operate concurrently. This interpretation is consistent with observational evidence that uncontrolled symptom relief rates following full low FODMAP diet restriction, reintroduction, and personalization reach at least 50% of patients, underscoring the importance of the structured delivery framework [11].

Similarly, the starch- and sucrose-reduced diet was non-inferior to the low FODMAP diet in a 155-patient RCT [35]. In contrast, a small pilot RCT of a Mediterranean diet, an outlier in the present analysis, suggested possible equivalence or even superiority for some outcomes. However, its small sample size limits firm conclusions [37].

Clinical Implications

The CARIBS trial, a well-designed single-blind RCT, demonstrated that both dietary interventions, including a low FODMAP diet combined with traditional IBS dietary advice, and optimized pharmacological treatment achieved clinically relevant reductions in IBS-SSS, with dietary arms showing a comparably large effect size to medication [45]. This trial is particularly relevant to the primary and secondary care context of the present review, supporting the potential positioning of dietary therapy as an effective first-line strategy. However, because the CARIBS trial did not directly measure medication escalation or subsequent medication use, the inference that dietary therapy reduces reliance on pharmacological treatment rests on comparable symptom improvement rather than a directly demonstrated outcome, and it warrants confirmation in trials specifically designed to assess this endpoint.

Strengths

Strengths of this review include its restriction to randomized trials, a comparator-stratified design that separates passive, guideline-aligned, mechanistic, and other active comparators, a pre-specified primary outcome and outcome hierarchy, a risk-of-bias assessment with RoB 2, and sensitivity analyses to explore sources of heterogeneity.

Limitations

The considerable heterogeneity observed in several subgroups, most notably the active comparator subgroup for IBS-SSS (I² = 95%), may reflect the diversity of dietary comparators included under the non-NICE category. This is likely, in part, an unavoidable feature of a field in which emerging dietary strategies (Mediterranean, starch- and sucrose-reduced diet, gut-directed hypnotherapy diets, and Ayurvedic diets) are increasingly trialed against the LFD. However, it may also reflect our categorization and pooling of clinically diverse interventions within a single active-comparator subgroup, a methodological choice in this review that could have contributed to the observed heterogeneity independent of the underlying literature. Sensitivity analyses identifying Jeitler et al. [32] and Singh et al. [37] as primary sources of heterogeneity are consistent with their respective methodological distinctions and markedly divergent effect estimates. This review also examined multiple comparator subgroups, outcome domains, and sensitivity analyses. However, no formal statistical adjustment for multiplicity was applied; the number of comparisons increases the likelihood that isolated or borderline statistically significant findings, such as the modest bloating benefit observed against active comparators, could arise by chance and should be interpreted accordingly.

The risk of bias assessment identified deviations from intended interventions as the most pervasive limitation, with 19 of 23 studies at high risk in this domain, an intrinsic limitation of all dietary intervention trials given the impossibility of dietary masking in open-label designs. This concern is shared across the literature; the 2024 Lancet network meta-analysis similarly rated all dietary evidence as low or very low confidence, in part because participants cannot be blinded [43]. Critically, unblinded dietary trials are susceptible to performance bias, nocebo effects, and differential expectations, which may inflate or attenuate estimated treatment effects. These limitations should temper the certainty of pooled estimates, particularly in subgroups dominated by high-risk studies.

Several limitations must be acknowledged. First, because participants cannot be blinded to dietary allocation in dietary intervention trials, performance and detection bias cannot be excluded, potentially inflating apparent treatment effects of the low FODMAP diet, particularly in comparisons against passive or habitual diet controls. Second, including heterogeneous active comparator interventions under a single subgroup introduces substantial clinical and methodological diversity that statistical models cannot fully resolve, as shown by the near-maximal I² values. Third, the meta-analysis relies on published aggregate data and therefore cannot conduct individual patient data analyses, which would permit exploration of effect modification by IBS subtype (IBS-D, IBS-C, IBS-M), baseline symptom severity, and method of dietary delivery. Fourth, included trials were predominantly short-term (weeks), and the long-term efficacy and safety of the low FODMAP diet, including its effects on nutritional adequacy and gut microbiota composition, remain incompletely characterized. Evidence indicates that prolonged FODMAP restriction can reduce the abundance of beneficial gut bacteria, including *Bifidobacterium* species, and may adversely affect microbial diversity [43]. Fifth, included studies measured and reported dietary adherence inconsistently, making it difficult to disentangle the effects of true dietary compliance from intention-to-treat exposure. Sixth, the applicability of the findings to primary care is uncertain, as most included trials were conducted in specialist secondary or tertiary care centers with access to registered dietitian support, which may not reflect routine clinical practice. Seventh, small sample sizes in several subgroups, particularly the two-study higher FODMAP comparison (n = 47), limit the reliability of pooled estimates and should be interpreted with caution. Finally, publication bias and small-study effects could not be formally assessed; no pooled comparison included 10 or more studies, the minimum recommended in the Cochrane Handbook for reliable funnel plot asymmetry detection and for Egger's test to have adequate statistical power, and funnel plots were therefore not presented, as visual inspection under these conditions risks misleading interpretation. Publication bias or small-study effects cannot be excluded. The certainty of evidence was not formally graded; conclusions should therefore be regarded as provisional.

Future Research

Future studies should test whether components shared by structured dietary interventions (education, engagement, and therapeutic support) contribute to treatment response, for example, by holding the level of professional support constant while varying dietary composition, and should assess long-term outcomes, nutritional adequacy, medication use, and predictors of individual response.

## Conclusions

This meta-analysis of 23 trials (2,164 participants) shows that the estimated relative effect of the low FODMAP diet varies substantially by comparator intervention. Against traditional dietary advice or habitual diet, it produces significant symptom improvement and quality-of-life gains. However, when compared with other structured dietary interventions, it showed no significant advantage for most outcomes, although a modest benefit for bloating was observed.

Our findings suggest that common components of structured dietary care, rather than the specific diet chosen, contribute to treatment response. Clinicians should therefore select dietary approaches based on individual patient preferences, tolerability, and access to dietitian support, rather than assuming that the low FODMAP diet is universally superior. Future research should identify predictors of individual response and conduct rigorous long-term comparisons of structured dietary strategies to advance personalized IBS management.

## Appendices

**Table 2:** Database-specific search strategies, search dates, and filters applied All electronic databases were searched from inception to 20 May 2026. IBS: irritable bowel syndrome, IBS-SSS: IBS symptom severity score, FODMAP: fermentable oligosaccharides, disaccharides, monosaccharides, and polyols, RCT: randomized controlled trial

Search terms	Database	Number of retrieved articles	Last search date	Database-specific filters applied		Number of articles	Causes for exclusion
The following search terms were used: ("irritable bowel syndrome" OR "IBS" OR "functional bowel disorder") AND ("low FODMAP" OR "FODMAP diet" OR "fermentable oligosaccharides" OR "fermentable carbohydrate*") AND ("symptom*" OR "abdominal pain" OR "bloating" OR "quality of life" OR "IBS-SSS") AND ("randomised controlled trial" OR "randomized controlled trial" OR "clinical trial")	PubMed	84	20 May 26	Article type: RCT; species: humans; language: English	Included after removal of duplications	1172	Automatic duplicate removal (338)
-	ScienceDirect	640	20 May 26	Article type: research articles (reviews excluded); language: English	Included after removal of reviews	1172	Automatic review removal (0)
-	Web of Science (WOS)	136	20 May 26	Document type: article; language: English	Included after abstract screening	345	-
-	Scopus	252	20 May 26	Document type: article; language: English	Included after full-text screening, i.e., for extraction	23	A total of 322 records were excluded during full-text screening. Duplicate publications, secondary analyses, and sub-analyses of existing trials accounted for the largest proportion of exclusions (N = 144). Ineligible study designs, including reviews, meta-analyses, study protocols, non-randomized trials, qualitative studies, uncontrolled pre-post designs, and case reports, were the second most common reason (N = 59). Studies enrolling populations without confirmed IBS, including those with inflammatory bowel disease, coeliac disease, functional heartburn, gastro-esophageal reflux disease, functional dyspepsia, or healthy volunteers, were excluded (N = 35). Publications available only as conference abstracts or without sufficient data for extraction were also excluded (N = 21). Further exclusions were applied to crossover trials without separable primary-period data (N = 11), studies reporting irrelevant outcomes (N = 11), trials using non-dietary comparators such as pharmacotherapy or behavioral therapy (N=9), inaccessible full texts (N = 9), trials comparing low-FODMAP diet plus a supplement against low-FODMAP diet alone without an independent dietary comparator (N = 6), studies in which the low-FODMAP diet was not the primary intervention, including reintroduction, challenge, and dietary adherence app designs (N = 13), and post-hoc metabolomic or microbiological secondary analyses published in non-English languages (N = 4).
-	MEDLINE (Ovid)	70	20 May 26	Publication type: RCT; limit: humans; language: English	-	-	-
-	Cochrane CENTRAL	298	20 May 26	Language: English; no additional database-specific filter applied (trial registry)	-	-	-
-	ClinicalTrials.gov	30	20 May 26	Study type: interventional studies; language: English	-	-	-
-	Grey literature	0	20 May 26	No database filter applied; conference proceedings, theses/dissertations, and trial registries hand-searched; language: English	-	-	-
-	Manual (reference-list search)	0	20 May 26	Not applicable - manual screening of reference lists of included and related articles	-	-	-
-	Total	1510	-	-	-	-	-
